# Drawer Algorithm: A New Metaheuristic Approach for Solving Optimization Problems in Engineering

**DOI:** 10.3390/biomimetics8020239

**Published:** 2023-06-06

**Authors:** Eva Trojovská, Mohammad Dehghani, Víctor Leiva

**Affiliations:** 1 Department of Mathematics, Faculty of Science, University of Hradec Králové, 500 03 Hradec Králové, Czech Republic; mohammad.dehghani@uhk.cz; 2 School of Industrial Engineering, Pontificia Universidad Católica de Valparaíso, Valparaíso 2362807, Chile; victor.leiva@pucv.cl

**Keywords:** drawer, exploitation, exploration, human-inspired methods, optimization

## Abstract

Metaheuristic optimization algorithms play an essential role in optimizing problems. In this article, a new metaheuristic approach called the drawer algorithm (DA) is developed to provide quasi-optimal solutions to optimization problems. The main inspiration for the DA is to simulate the selection of objects from different drawers to create an optimal combination. The optimization process involves a dresser with a given number of drawers, where similar items are placed in each drawer. The optimization is based on selecting suitable items, discarding unsuitable ones from different drawers, and assembling them into an appropriate combination. The DA is described, and its mathematical modeling is presented. The performance of the DA in optimization is tested by solving fifty-two objective functions of various unimodal and multimodal types and the CEC 2017 test suite. The results of the DA are compared to the performance of twelve well-known algorithms. The simulation results demonstrate that the DA, with a proper balance between exploration and exploitation, produces suitable solutions. Furthermore, comparing the performance of optimization algorithms shows that the DA is an effective approach for solving optimization problems and is much more competitive than the twelve algorithms against which it was compared to. Additionally, the implementation of the DA on twenty-two constrained problems from the CEC 2011 test suite demonstrates its high efficiency in handling optimization problems in real-world applications.

## 1. Introduction

Optimization is the process by which the best possible solution to a problem is identified. Each optimization problem can be modeled using three main components: variables, constraints, and objective functions [1]. With the advancement of science and technology, optimization problems have become more complex and require effective methods. Stochastic algorithms are effective methods for solving optimization problems by randomly scanning the search space and using random operators. These algorithms first generate a population of solvable solutions to a given problem and then improve those solutions in an iterative process to eventually converge on a suitable solution [2]. 

The best solution to an optimization problem is called the global optimum. However, there is no guarantee that the algorithms will precisely provide such an optimum. For this reason, the solution obtained from the optimization algorithm for a problem is called a quasi-optimal, which may or may not be equal to the global optimum [3].

Scientists have introduced numerous algorithms to provide better quasi-optimal solutions to optimization problems than the existing algorithms. These optimization algorithms are employed in various fields in the literature, such as training an artificial neural network [4,5], cyber-physical systems [6], energy and energy carriers [7,8,9], and electrical engineering [10,11,12,13,14,15], to solve the problem and achieve a better quasi-optimal solution. 

With the advancement of computer processing capabilities in recent years, there has been a chance to study engineering problems more precisely and thoroughly, considering numerous uncertainties and constraints. Assuming diverse constraints and increasing the number of variables in various engineering challenges necessitates using more powerful problem-solving approaches. Metaheuristic algorithms are effective tools for modern researchers and engineers, as seen in numerous implementations [16]. Metaheuristic algorithms could be employed if an engineering problem can be algorithmically described, precisely defined, and parameterized. Solving real-world problems very often requires connecting several disciplines within the optimization procedure. The advances in metaheuristic algorithm studies continuously push the boundary of application feasibility, making the optimization processes more efficient and accurate. Metaheuristic algorithms are higher-level tools that can positively and decently impact any engineering problem [17].

Based on their fundamental design ideas, optimization algorithms can be divided into five groups: swarm-based, evolutionary-based, physics-based, game-based, and human-based approaches.

Swarm-based optimization algorithms are based on modeling observations in nature, behavior, and the life of animals, insects, and other living organisms. Particle swarm optimization (PSO) is a widely used algorithm based on simulating the behavior of a group of birds and fish [18]. The ant colony optimization (ACO) algorithm has been developed considering the behavior of ants in finding the shortest path to food sources [19]. The artificial bee colony (ABC) algorithm [20] is a nature-inspired optimization approach inspired by the collective behavior and intelligent foraging of honey bee colonies.

The krill herd (KH) algorithm simulates the herding behavior of krill [21]. Gray wolf optimization (GWO) is developed by simulating the natural behavior of gray wolves in hierarchical prey hunting [22]. The egret swarm optimization algorithm (ESOA) was proposed based on the simulation of two egret species’ hunting behavior (great egret and snowy egret) [23]. The beetle antennae search (BAS) was inspired by the food-foraging behavior of beetles [24]. Some other swarm-based approaches are the serval optimization algorithm (SOA) [25], subtraction-average-based optimizer (SABO) [26], marine predators algorithm (MPA) [27], coati optimization algorithm (COA) [28], tunicate swarm algorithm (TSA) [29], orchard algorithm (OA) [30], social spider optimizer (SSO) [31], emperor penguin optimizer (EPO) [32], lion optimization algorithm (LOA) [33], cuckoo search (CS) [34], green anaconda optimizer (GOA) [35], two-stage optimizer (TSO) [36], manta ray foraging optimizer (MRFO) [37], mountain gazelle optimizer (MGO) [38], and two-stage improved gray wolf optimizer (IGWO) [39]. 

Evolutionary-based optimization algorithms are introduced using random and evolutionary operators such as selection and simulation of genetic and biological sciences. The genetic algorithm (GA), one of the most famous optimization approaches to date, belongs to this group. In the design of the GA, modeling of the reproductive process with selection, crossover, and mutation operators is used [40]. Some other evolutionary algorithms are evolution strategy (ES) [41], biogeography-based optimizer (BBO) [42], genetic programming (GP) [43], and differential evolution (DE) [44].

Physics-based optimization algorithms are inspired by various physical laws and phenomena. The simulated annealing (SA) algorithm is one of the oldest physics-based methods, introduced according to the modeling of the metal melting process in metallurgy. The gravitational search algorithm (GSA) is based on gravitational force modeling and Newtonian laws of motion [45]. The turbulent flow of water-based optimization (TFWO) [46] is designed on abnormal oscillations in water turbulent flow. The thermal exchange optimizer (TEO) [47] is a technique that draws inspiration from Newton’s law of cooling. Some other physics-based approaches are the galaxy-based search algorithm (GbSA) [48], black hole (BH) [49], ray optimizer (RO) [50], big bang-big crunch (BB-BC) [51], small world optimization algorithm (SWOA) [52], magnetic optimization algorithm (MOA) [53], and artificial chemical reaction optimization algorithm (ACROA) [54].

Game-based optimization algorithms are developed according to the relationships and behavior of players in games, game rules, coaches’ movements, and referees. For example, the league championship algorithm (LCA) [55] and football game-based optimizer (FGBO) [56] are based on simulating players’ behavior and the interactions of football clubs during a tournament season. The volleyball premier league (VPL) algorithm was developed based on interaction of volleyball teams during one season [57]. Some other game-based algorithms are an improved football game optimizer (IFGO) [58], the running city game optimizer (RCGO) [59], Nash game-efficient global optimizer (Nash-EGO) [60], and a generalized soccer league optimizer (SLO) [61].

Human-based optimization algorithms are introduced and inspired by humans’ behaviors, choices, thoughts, decisions, and other strategies in their personal and social life. The teaching-learning-based optimizer (TLBO) [62] can be mentioned as one of this group’s most famous and widely used algorithms. TLBO is designed based on modeling communication and educational interactions between teachers and students, as well as students with each other in the classroom environment, to improve the level of classroom knowledge. Therapeutic interactions between doctors and patients to examine and treat patients are employed in the design of the doctor and patient optimizer (DPO) [63]. The process of voting in elections has been the main idea in the design of the election based optimization algorithm (EBOA) [64]. Some other human-based approaches are the teamwork optimization algorithm (TOA) [65], driving training-based optimizer (DTBO) [66], archery algorithm (AA) [67], group optimizer (GO) [68], and following optimization algorithm (FOA) [69].

The main research question is whether, once new metaheuristic algorithms have been designed, is there still a need to introduce a newer algorithm to deal with optimization problems or not? In response to this question, the no free lunch (NFL) [70] theorem explains that the high success of a particular algorithm in solving a set of optimization problems will not guarantee the same performance of that algorithm in other optimization problems. It cannot be assumed that implementing an algorithm on an optimization problem will necessarily lead to successful results. According to the NFL theorem, no metaheuristic algorithm is the best optimizer for solving all the corresponding problems. The NFL theorem motivates researchers to search for better solutions for optimization problems by designing newer metaheuristic algorithms. The NFL theorem has also inspired the authors of this article to provide more effective solutions in dealing with optimization problems by creating a new metaheuristic algorithm.

The novelty and innovation of the present study lie in designing a new human-based approach called the drawer algorithm (DA) for optimization applications. The main contributions of this article are stated as follows:

- A new metaheuristic algorithm is presented, motivated by people maintaining order in commode drawers.
- The DA is modeled by simulating the process of selecting the appropriate objects from different drawers to create an optimal combination.
- The DA’s performance is tested on fifty-two benchmark functions of unimodal, high-dimensional, fixed-dimensional multimodal types and the CEC 2017 test suite.
- The DA’s results are compared with the performance of twelve well-known metaheuristic algorithms.
- The efficiency of the DA in handling real-world applications is tested on twenty-two constrained optimization problems from the CEC 2011 test suite.

The rest of the article is organized as follows. In Section 2, the DA is introduced and modeled. Then, the simulation studies are presented in Section 3. Next, the performance of the DA in solving real-world applications is evaluated in Section 4. Finally, conclusions and suggestions for further studies in line with this work are provided in Section 5.

## 2. Drawer Algorithm

This section introduces the DA and its mathematical model. As mentioned, the main inspiration for designing the DA is to simulate the process of selecting objects from the drawers of a cupboard to form an optimal combination. In the DA, it is assumed that several drawers are available, each containing a certain number of objects. One object must be picked from each drawer to create a proper combination of objects inside the drawers. Picking up the appropriate objects from the drawers and putting them together is an optimization process that can inspire the design of an algorithm.

### 2.1. Originality of the DA

Human-based algorithms were presented in the introduction section as one of the classes of optimizers in grouping based on the source of inspiration in design. Many human behaviors are intelligent processes that can be the basis for designing new optimization algorithms. One of the intelligent behaviors of humans in life is their attempt to pick up the objects they want from the drawers of a closet. For example, this can be to choose a suitable style of clothing. In this case, it is assumed that in each drawer of the cabinet, a person faces different choices for each type of clothing: a drawer for watches, a drawer for jewelry, a drawer for shoes, a drawer for ties, a drawer for hats, etc. Therefore, choosing a suitable clothing style from cabinet drivers is a smart strategy with extraordinary potential for designing an optimization algorithm. Based on the best knowledge obtained from the literature review, the originality of the proposed approach was confirmed because no optimization algorithm has been designed based on modeling the strategy of humans in choosing objects from closet drawers. Thus, to address such a research gap, in this paper, a human-based optimizer called DA has been developed based on the mathematical modeling of human strategy in selecting the desired objects from the wardrobe drawers. The unique characteristics of the DA are as follows:

- In the design of the DA, the strong dependence of the population update process on specific members of the population, such as the best member, is avoided. This feature makes it possible to prevent the algorithm from getting stuck in local optima by increasing the ability to search for places exploring and directing the algorithm to the main optimal area in the search space.
- Stagnation in optimization algorithms occurs when all population members are gathered at the same position. In this case, all members of the population become similar. If the algorithm cannot remove the population from this condition, the update process will not be successful. In the DA design, using a random combination of population members in the updating process by making extensive changes in the position of the members can bring the algorithm out of the static state.
- In the design of the DA, it is assumed that the number of objects inside the drawers decreases during successive iterations of the algorithm. These conditions lead to a balance between exploration and exploitation in the search space. At the beginning of the implementation of the algorithm, the number of objects is at its maximum, which can lead to large changes in the position of the population members with the possibility of making more combinations of drawers. Hence, in the initial iterations, priority is given to global search and exploration as an all-around search in the problem-solving space to identify the main optimal region. Then, by increasing the iterations of the algorithm, the number of objects inside the drawers decreases, resulting in fewer combinations of drawers. These conditions lead to limited movements in the position of population members. Therefore, by increasing the iterations of the algorithm, priority is given to local search and exploitation so that the algorithm converges toward better solutions in promising areas.

### 2.2. Mathematical Modeling

The DA is a metaheuristic approach that solves optimization problems iteratively. During each iteration, the population members of the DA scan the search space of the problem to converge toward the optimal solution. 

The population of the algorithm can be modeled using a matrix that is specified as

$$X={\left[\begin{array}{c} {\vec{X}}_{1}\\ \vdots \\ {\vec{X}}_{i}\\ \vdots \\ {\vec{X}}_{N} \end{array}\right]}_{N\times m}={\left[\begin{array}{ccccc} x_{1,1} & \cdots & x_{1,j} & \cdots & x_{1,m}\\ \vdots & \ddots & \vdots & ⋰ & \vdots \\ x_{i,1} & \cdots & x_{i,j} & \cdots & x_{i,m}\\ \vdots & ⋰ & \vdots & \ddots & \vdots \\ x_{N,1} & \cdots & x_{N,j} & \cdots & x_{N,m} \end{array}\right]}_{N\times m},$$

where X is the population matrix of the DA, N is the number of population members, m is the number of variables, ${\vec{X}}_{i}=\left(x_{i,1},\ldots ,x_{i,j},\ldots ,x_{i,m}\right),$ with i=1,…,N being the ith proposed solution, and $x_{i,j}$ being its jth component (the jth variable of the problem). In the beginning, all population members must be randomly initialized by means of

(1) $$x_{i,j}=lb_{j}+\mathrm{rand}\left(0,1\right)\cdot \left(ub_{j}-lb_{j}\right),\;i=1,\ldots ,N,\;j=1,\ldots ,m,$$

where $x_{i,j}$ is the value of the jth variable determined by the ith DA’s member $X_{i}$, the function $\mathrm{rand}\left(0,1\right)$ generates uniformly a random number in the interval $\left[0,\;1\right]$, whereas $lb_{j}$ and $ub_{j}$ are the lower and upper bounds of the jth problem variable, respectively.

Based on the population of the algorithm that is the proposed solution to the given problem, the objective function F (with m variables) can be evaluated. The values obtained for the objective function are shown using an expression given by

(2) $$\vec{F}={\left[\begin{array}{c} F_{1}\\ \vdots \\ F_{i}\\ \vdots \\ F_{N} \end{array}\right]}_{N\times 1}={\left[\begin{array}{c} F\left({\vec{X}}_{1}\right)\\ \vdots \\ F\left({\vec{X}}_{i}\right)\\ \vdots \\ F\left({\vec{X}}_{N}\right) \end{array}\right]}_{N\times 1},$$

where $\vec{F}$ is the vector of the obtained objective function values, and $F_{i}$ is the value of the objective function for the ith proposed solution. Thus, $F_{i}=F\left(x_{i,1},\ldots ,x_{i,j},\ldots ,x_{i,m}\right),$ with i=1,…,N.

The main concept behind updating the population matrix in the DA is to utilize a carefully selected combination of drawers containing variable information. Specifically, the DA assumes the presence of a commode with the same number of drawers as variables exist in the optimization problem. Each drawer in the commode contains different suggested values for the corresponding variable. The commode and drawers can be mathematically modeled using the equations formulated as

(3) $$D={\left[\begin{array}{c} {\vec{D}}_{1}\\ \vdots \\ {\vec{D}}_{j}\\ \vdots \\ {\vec{D}}_{m} \end{array}\right]}_{m\times 1}={\left[\begin{array}{ccccc} d_{1,1} & \cdots & d_{1,k} & \cdots & d_{1,N_{D}}\\ \vdots & \ddots & \vdots & ⋰ & \vdots \\ d_{j,1} & \cdots & d_{i,k} & \cdots & d_{i,N_{D}}\\ \vdots & ⋰ & \vdots & \ddots & \vdots \\ d_{m,1} & \cdots & d_{m,k} & \cdots & d_{m,N_{D}} \end{array}\right]}_{m\times N_{D}\left(t\right)},$$

(4) $$N_{D}\left(t\right)=\lceil \left(1-\frac{t}{T}\right)\cdot N\rceil ,\;t=1,\ldots ,T,$$

(5) $${\vec{D}}_{j}=\left(x_{\mathrm{rand}\left(N\right),j}\left|\;k=1,2,\ldots ,N_{D}\left(t\right)\right.\right),\;j=1,\ldots ,m\;,$$

where D is the drawer matrix, ${\vec{D}}_{j}$ is the vector of values in the jth drawer, for j=1,…,m, ⌈·⌉ represents the usual mathematical ceiling function, T is the total number of iterations, $N_{D}\left(t\right)$ is the number of drawers in the tth iteration, and $x_{\mathrm{rand}\left(N\right),j}$ is the corresponding element of the ith column of the $\mathrm{rand}\left(N\right)$th row, where $\mathrm{rand}\left(N\right)$ is the random function, which generates uniformly a random number from the set $\left\{1,\ldots ,\;N\right\}$.

Metaheuristic algorithms based on random search in the corresponding space are able to find suitable solutions for optimization problems. Additionally, to provide effective search, metaheuristic algorithms must be able to scan the search space well at two levels: (i) globally with the concept of exploration and (ii) locally with the concept of exploitation.

In the DA design, choosing a random combination and using it in the update process leads to large population displacements in the search space and thus increases the exploration power. In addition, in the DA design, the number of proposed values for each variable in each drawer decreases according to Equation (4) during the iterations of the algorithm. This leads to smaller displacements increasing the exploitation power of the algorithm.

Equation (4) is selected so that, in the initial iterations, the number of suggested values for each variable is at the maximum value to increase exploration. Then, it decreases during the iterations of the algorithm to prioritize the exploitation ability. Therefore, in the DA design, the balance has been established between exploration and exploitation during the iterations of the algorithm.

In the DA, a random combination created by values from drawers is used to update each member of the population. This random combination directs the population members into the search space. The process of forming a random combination of drawers is such that exactly one value is selected from each drawer, which is considered the value of a problem variable. Then, these selected values from the drawers together produce a random combination to guide the population member. The process of forming this random combination is presented as

(6) $${\vec{C}}_{i}=\left\{d_{j,\mathrm{rand}\left(N_{D}\left(t\right)\right)}\;\left|\;j=1,\ldots ,m\right.\right\},\;i=1,\ldots ,N,$$

where ${\vec{C}}_{i}$ is the random combination to guide the ith population member, $c_{i,j}$ is its jth dimension, and $d_{j,\mathrm{rand}\left(N_{D}\left(t\right)\right)}$ is the corresponding element of the jth row of the $\mathrm{rand}\left(N_{D}\left(t\right)\right)$th column of the matrix D, with $\mathrm{rand}\left(N_{D}\left(t\right)\right)$ being a function that generates a random number from the set $\left\{1,\;\ldots ,\;N_{D}\left(t\right)\right\}$.

**Figure 1:**
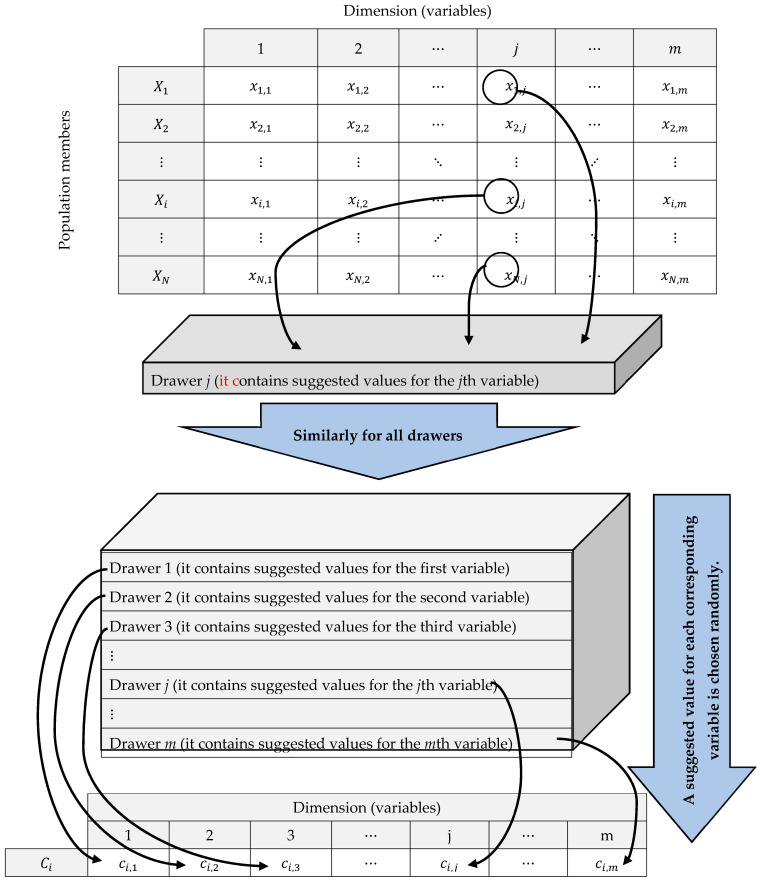
DA schematic: Forming drawers and random combination construction for population update.

After determining the random composition, each population member is updated in the search space using the expressions presented as

(7) $$x_{i,j}^{\mathrm{new}}=\left\{\begin{array}{cc} x_{i,j}+\mathrm{rand}\left(0,1\right)\cdot \left(c_{i,j}-\mathrm{rand}\left(2\right)\cdot x_{i,j}\right), & F_{i}^{C}<F_{i},\\ x_{i,j}+\mathrm{rand}\left(0,1\right)\cdot \left(x_{i,j}-c_{i,j}\right), & \mathrm{else}, \end{array}\right.$$

(8) $$X_{i}=\left\{\begin{array}{cc} X_{i}^{\mathrm{new}}, & F_{i}^{\mathrm{new}}\leq F_{i},\\ X_{i},\; & \mathrm{else}, \end{array}\right.$$

where $X_{i}^{\mathrm{new}}$ is the new status of the ith proposed solution, $x_{i,j}^{\mathrm{new}}$ is its jth dimension, $F_{i}^{\mathrm{new}}$ is its objective function value, and $F_{i}^{C}$ is the objective function of random combination to guide the ith population member. The process of forming the drawers, as well as the way of creating a random combination to guide each population member, is shown as a schematic representation in Figure 1.

**Figure 2:**
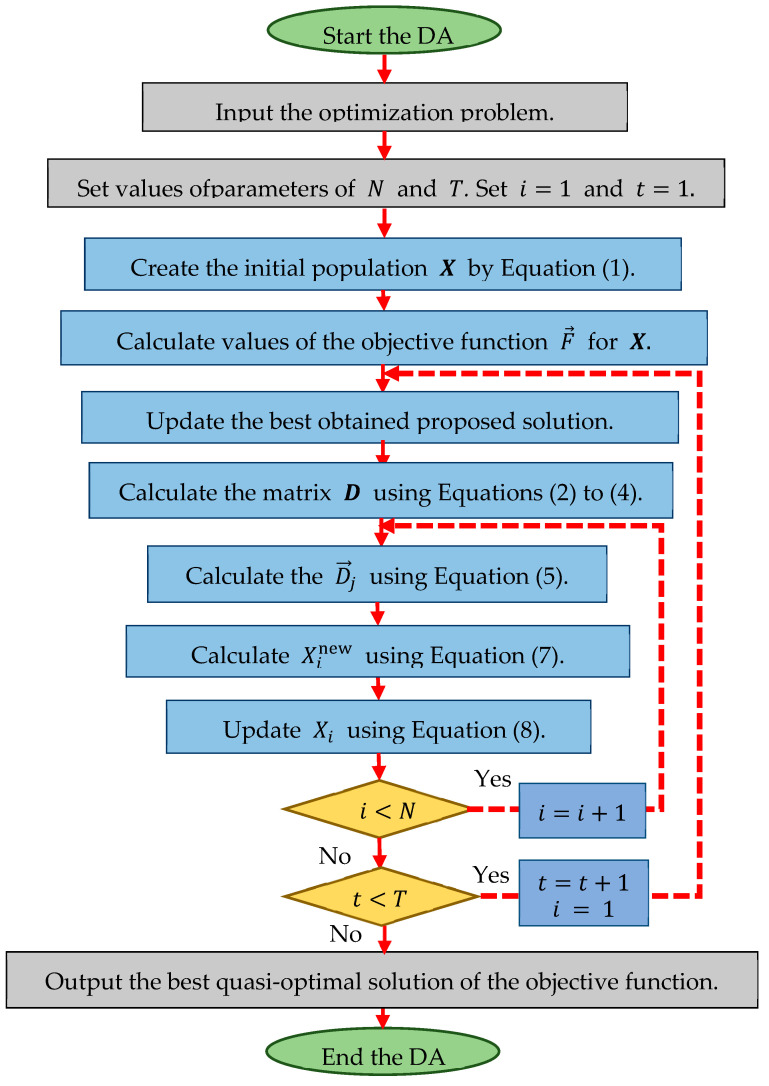
Flowchart of the DA.

After updating the population, an iteration of the algorithm is completed. The process of updating the algorithm population continues until the end of its iterations, according to Equations (4)–(8). Algorithm 1 presents the pseudo-code of the DA, and Figure 2 shows the corresponding flowchart.
**Algorithm 1.** Pseudocode of the DA.Start DA.1.Input: the optimization problem.2.Set the number of iterations T and the number of members of the population N.3.Generate the initial population X at random by Equation (1).4.Evaluate the initial population X (compute $\vec{F}$ by Equation (2)).5.For t=1:T 6.
 Update the best proposed solution.7.
 Calculate the drawer matrix based on Equations (3)–(5).8.
 For i=1:N 9.
 Generate a random combination based on Equation (6).10.
 Calculate a new status of population member based on Equation (7).11.
 Update the ith population member using Equation (8).12.
 end13.
 Save the best proposed solution so far.14.end15.Output: the best obtained proposed solution.End DA.

### 2.3. Computational Complexity

Based on the DA phases and implementation steps, the computational complexity of the proposed approach is as follows: DA initialization for solving an optimization problem based on *m* decision variables has a complexity of $O\left(Nm\right)$, where N is the number of search agents. Furthermore, updating search agents has a complexity equal to $O\left(NmT\right)$, with T being the total number of iterations of the algorithm. Therefore, the total computational complexity of the DA is equal to $O\left(Nm\left(1+T\right)\right)$.

## 3. Simulation Studies and Results

In this section, the ability of the DA to solve optimization problems is studied. Various objective functions of unimodal, high-dimensional multimodal, and fixed-dimensional multimodal types and the CEC 2017 test suite [71] have been used to evaluate the proposed approach. The DA is compared with twelve algorithms: GA, PSO, GSA, TLBO, GWO, MVO, WOA, MPA, TSA, RSA, WSO, and AVOA. The values of the control parameters of the competitor algorithms are specified in Table 1. 

The DA and each of the competing algorithms were implemented in twenty independent runs on the benchmark functions, where each independent run consisted of 1000 iterations. Optimization results were reported using six indicators: mean, best, worst, standard deviation (std), median, and rank. The ranking criterion for metaheuristic algorithms was based on providing a better value for the mean index.

### 3.1. Evaluation of Unimodal Functions

The results of optimizing the unimodal objective functions using the DA and twelve other algorithms are presented in Table 2. Based on the optimization results, the DA provided the best solution to the problem, that is, global optima, when solving F1, F2, F3, F4, F5, F6, and F7. The simulation results show that the DA significantly outperforms the other twelve algorithms. 

### 3.2. Evaluation of High-Dimensional Multimodal Functions 

The DA and twelve other algorithms were implemented on the functions F8 to F13, and the results are presented in Table 3. The analysis of this table shows that the DA provides the optimal solution for F9 and F11. The DA is also the best optimizer for solving F8, F10, F12, and F13. The optimization results indicate the superiority of the DA compared to the twelve other algorithms.

### 3.3. Evaluation of Fixed-Dimensional Multimodal Functions 

**Figure 3:**
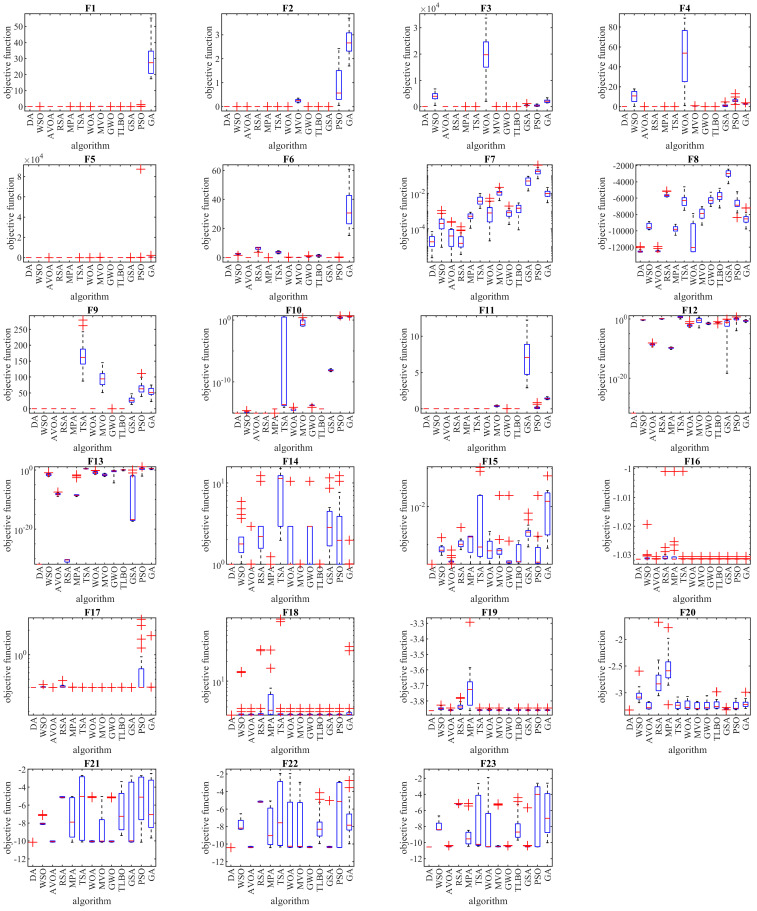
Boxplots of the performance of the DA and competitor algorithms on F1 to F23 test functions.

**Figure 4:**
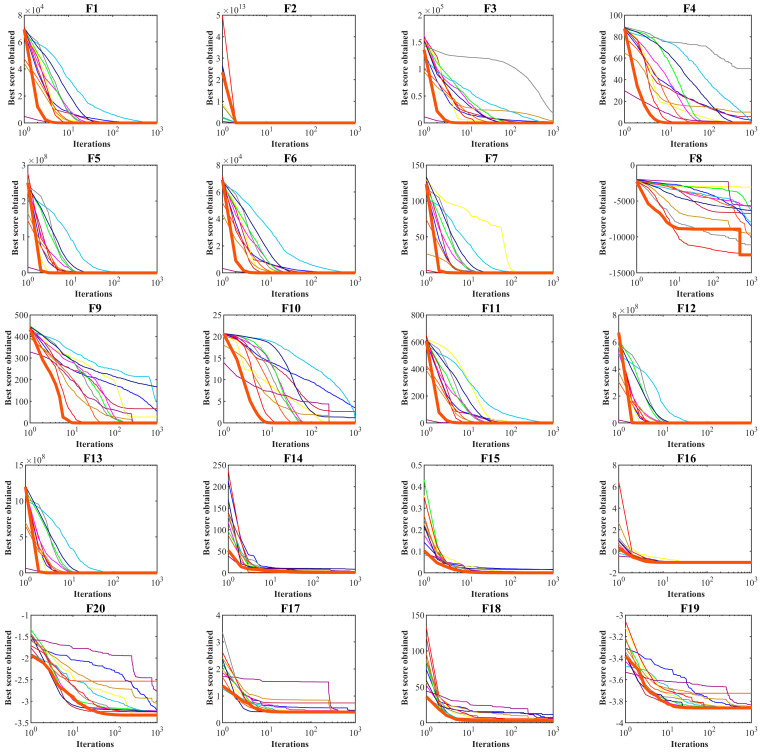

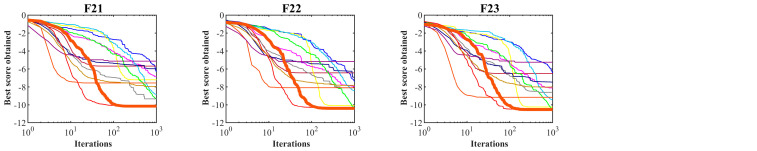
Convergence curves of the DA and competitor algorithms on F1 to F23 test functions.

The results of optimizing F14 to F23 functions using the DA and twelve compared algorithms are presented in Table 4. This table shows that the DA presents the global optimum for F17. The DA is the best optimizer in solving F14, F15, F16, F18, F19, F20, F21, F22, and F23. Comparing the performance of optimization algorithms against DA indicates the high ability of the DA to solve multimodal problems. The performance of the DA and competitor algorithms in solving functions F1 to F23 is presented in the boxplots of Figure 3. The convergence curves of the DA and competing algorithms while solving algorithm iterations are drawn in Figure 4.

### 3.4. Evaluation of the CEC 2017 Test Suite

**Figure 5:**
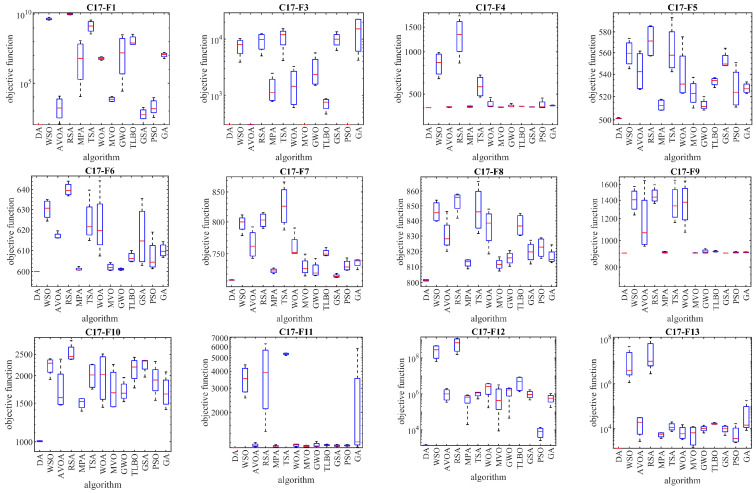

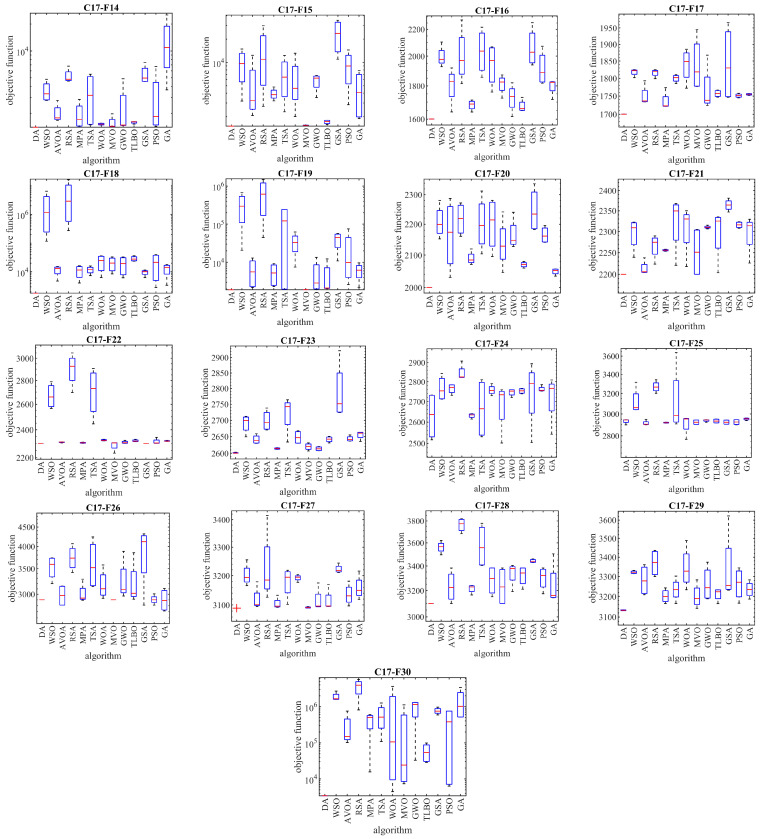
Boxplots of the performance of the DA and competitor algorithms on the CEC 2017 test suite.

Next, the evaluation of the DA approach in dealing with optimization tasks on the CEC 2017 test suite is discussed. This test suite has thirty standard benchmark functions, consisting of three unimodal functions C17-F1 to C17-F3, seven multimodal functions C17-F4 to C17-F10, ten hybrid functions C17-F11 to C17-F20, and ten hybrid functions C17-F21 to C17-F30. Of these functions, the C17-F2 function was excluded from the simulation studies due to its unstable behavior. Complete information on the CEC 2017 test suite is provided in [63]. The optimization results for the CEC 2017 test suite using DA and competitor algorithms are reported in Table 5. The boxplots obtained from the performance of the optimization algorithms in handling the CEC 2017 test suite are drawn in Figure 3. Based on the simulation results, the DA is the first best optimizer for C17-F1, C17-F3 to C17-F21, C17-F23, C17-F24, and C17-F27 to C17-F30. Analysis of the simulation results shows that the DA, by providing better results in most benchmark functions of the CEC 2017 test suite, indicated superior performance compared to competitor algorithms Figure 5.

### 3.5. Statistical Analysis

The simulation results based on the mean and standard deviation have already demonstrated the superior performance of the DA compared to the twelve competitor algorithms. 

Now, we conduct a statistical analysis to determine whether the superiority of the DA over the other twelve algorithms is statistically significant. To this end, the non-parametric Wilcoxon signed-rank test [72] is employed. The statistical analysis results are presented in Table 6, where a p-value indicates whether the difference in performance between the DA and a competitor algorithm is statistically significant. If a p-value is less than 0.05, then the DA shows an important advantage over the corresponding algorithm in terms of statistical significance.

## 4. DA for Real-World Applications

**Figure 6:**
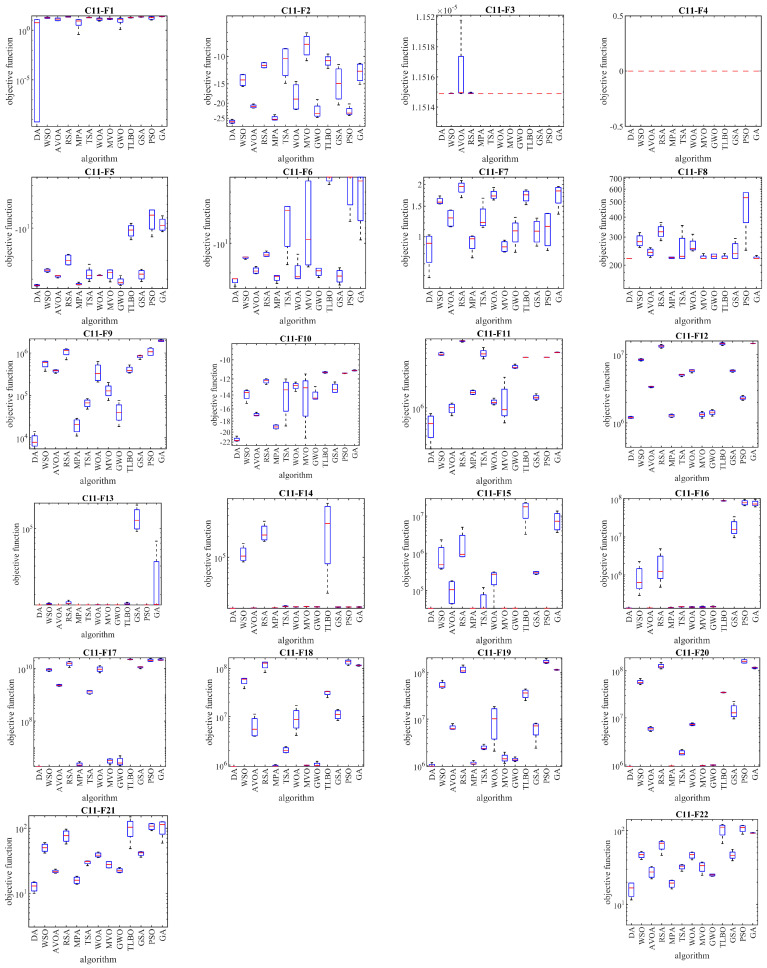
Boxplots of the performance of the DA and competitor algorithms on the CEC 2011 test suite.

In this section, we examine the effectiveness of the DA in tackling optimization problems in real-world applications. We apply the DA and competing algorithms to solve twenty-two optimization problems from the CEC 2011 test suite to accomplish this. These optimization problems include parameter estimation for frequency-modulated sound waves, the Lennard-Jones potential problem, the bifunctional catalyst blend optimal control problem, optimal control of a nonlinear stirred tank reactor, the Tersoff potential for the model Si (B), the Tersoff potential for the model Si (C), spread spectrum radar polyphase code design, transmission network expansion planning problem, large-scale transmission pricing problem, circular antenna array design problem, and the electronic logging device (ELD) problems (which consist of DED instance 1, DED instance 2, ELD instance 1, ELD instance 2, ELD instance 3, ELD instance 4, ELD instance 5, hydrothermal scheduling instance 1, hydrothermal scheduling instance 2, and hydrothermal scheduling instance 3), the Messenger spacecraft trajectory optimization problem, and the Cassini 2 spacecraft trajectory optimization problem. A full description of the CEC 2011 test suite is provided in [73]. The implementation results for the DA and competing algorithms on the CEC 2011 test suite are presented in Table 7. The boxplots obtained from the performance of the metaheuristic algorithms in solving this test suite are shown in Figure 6. Based on the simulation results, we find that the DA outperforms all other optimizers for C11-F1 to C11-F22. Analysis of the optimization results reveals that the DA, which provides better results for most optimization problems, demonstrates superior performance in handling the CEC 2011 test suite compared to the competing algorithms. Statistical analysis indicates that the superiority of the DA over competing algorithms is significant from a statistical standpoint. The simulation results demonstrate the high ability of the DA to handle optimization problems in real-world applications.

## 5. Conclusions and Future Works

This article proposed a new metaheuristic approach called the drawer algorithm to solve optimization problems effectively. The main inspiration for this algorithm comes from the simulation of bringing out objects from different commode drawers and creating a suitable combination of those objects. First, the drawer algorithm was introduced, and then its mathematical modeling was studied. The ability of the drawer algorithm in optimization was tested by solving fifty-two objective functions, including unimodal functions, high-dimensional multimodal functions, fixed-dimensional multimodal functions, and the CEC 2017 test suite. The results of optimizing the unimodal functions indicated the high exploitation power of the proposed algorithm in solving problems. The optimization of multimodal function results showed that the drawer algorithm provides suitable quasi-optimal solutions by striking a suitable balance between exploration and exploitation. In addition, the drawer algorithm was compared with the results for twelve well-known algorithms. The simulation results showed that the drawer algorithm has a higher ability to optimize than similar algorithms and is much more competitive. Furthermore, implementing the drawer algorithm on twenty-two constrained optimization problems from the CEC 2011 test suite demonstrated the proposed approach’s precise capability in dealing with real-world applications.

We offer suggestions for future work related to the design of binary and multi-objective versions of the drawer algorithm. Additionally, applications of the drawer algorithm to solve optimization problems in various cyber-physical systems and real-world problems to achieve optimal solutions are other suggestions for future research.

## Figures and Tables

**Table 1:** Control parameter values.

Algorithm	Parameter	Value
GA	Type	Real coded
	Selection	Roulette wheel (proportionate)
	Crossover	Whole arithmetic (probability=0.8, $\alpha \in \left[-0.5,\;1.5\right]$)
	Mutation	Gaussian (probability=0.05)
PSO	Topology	Fully connected
	Cognitive and social constant	$\left(C_{1},\;C_{2}\right)=\left(2,\;2\right)$
	Inertia weight	Linear reduction from 0.9 to 0.1
	Velocity limit	10% of dimension range
GSA	Alpha, $G_{0},\;R_{\mathrm{norm}},\;R_{\mathrm{power}}$	20, 100, 2, 1
TLBO	$T_{F}$: teaching factor	$T_{F}=\mathrm{round}\;\left[\left(1+\mathrm{rand}\right)\right]$
	Random number	rand is a random number between $\left[0,\;1\right]$
GWO	Convergence parameter (a)	a: Linear reduction from 2 to 0
MVO	Wormhole existence probability (WEP)	$\min\left(\mathrm{WEP}\right)=0.2$ and $\max\left(\mathrm{WEP}\right)=1$
	Exploitation accuracy over the iterations (p)	p=6
WOA	Convergence parameter (*a*)	*a*: Linear reduction from 2 to 0
	r is a random vector in $\left[0,\;1\right].$	
	l is a random number in $\left[-1,\;1\right].$	
TSA	$P_{\min}$ and $P_{\max}$	1, 4
	$c_{1},\;c_{2},\;c_{3}$	Random numbers lie in the range of $\left[0,\;1\right]$.
MPA	Constant number	P=0.5
	Random vector	*R* is a vector of uniform random numbers in $\left[0,\;1\right]$
	Fish aggregating devices (FADs)	FADs=0.2
	Binary vector	U=0 or 1
RSA	Sensitive parameter	β=0.01
	Sensitive parameter	α=0.1
	Evolutionary sense (ES)	ES: randomly decreasing values between 2 and −2
AVOA	$L_{1},\;L_{2}$	0.8, 0.2
	*w*	2.5
	$P_{1},\;P_{2},\;P_{3}$	0.6, 0.4, 0.6
WSO	$F_{\min}$ and $F_{\max}$	0.07, 0.75
	$\tau ,\;a_{0},\;a_{1},\;a_{2}$	4.125, 6.25, 100, 0.0005

**Table 2:** Optimization results for unimodal functions (F1–F8).

		DA	WSO	AVOA	RSA	MPA	TSA	WOA	MVO	GWO	TLBO	GSA	PSO	GA
F1	mean	0	2.70 × 10^−152^	0	0	1.86 × 10^−49^	4.51 × 10^−47^	1.30 × 10^−151^	0.145162	1.72 × 10^−59^	2.45 × 10^−74^	1.29 × 10^−16^	0.097939	29.59
	best	0	1.80 × 10^−171^	0	0	3.69 × 10^−52^	1.40 × 10^−50^	9.10 × 10^−171^	0.102355	1.45 × 10^−61^	5.69 × 10^−77^	5.20 × 10^−17^	4.72 × 10^−4^	17.39095
	worst	0	5.20 × 10^−151^	0	0	1.61 × 10^−48^	3.21 × 10^−46^	2.60 × 10^−150^	0.195279	7.48 × 10^−59^	2.52 × 10^−73^	3.63 × 10^−16^	1.355952	55.22585
	std	0	1.30 × 10^−151^	0	0	4.20 × 10^−49^	1.07 × 10^−46^	6.40 × 10^−151^	0.029675	2.29 × 10^−59^	6.58 × 10^−74^	7.65 × 10^−17^	0.332239	11.18532
	median	0	4.20 × 10^−160^	0	0	4.04 × 10^−50^	4.15 × 10^−48^	2.10 × 10^−159^	0.146027	1.04 × 10^−59^	1.64 × 10^−75^	1.10 × 10^−16^	0.009429	27.35582
	rank	1	2	1	1	6	7	3	10	5	4	8	9	11
F2	mean	0	4.90 × 10^−106^	1.10 × 10^−276^	0	6.76 × 10^−28^	2.05 × 10^−28^	2.40 × 10^−105^	0.251424	1.31 × 10^−34^	6.56 × 10^−39^	5.32 × 10^−8^	0.86873	2.705022
	best	0	1.50 × 10^−118^	1.30 × 10^−306^	0	1.79 × 10^−29^	1.96 × 10^−30^	7.70 × 10^−118^	0.155288	4.72 × 10^−36^	8.56 × 10^−40^	3.38 × 10^−8^	0.043928	1.69317
	worst	0	5.30 × 10^−105^	2.10 × 10^−275^	0	4.57 × 10^−27^	1.77 × 10^−27^	2.70 × 10^−104^	0.353611	7.67 × 10^−34^	2.37 × 10^−38^	1.20 × 10^−7^	2.418765	3.69274
	std	0	1.50 × 10^−105^	0	0	1.17 × 10^−27^	5.66 × 10^−28^	7.40 × 10^−105^	0.067341	2.09 × 10^−34^	5.97 × 10^−39^	2.00 × 10^−8^	0.772627	0.582406
	median	0	6.70 × 10^−109^	6.30 × 10^−290^	0	3.41 × 10^−28^	1.92 × 10^−29^	3.30 × 10^−108^	0.260325	6.31 × 10^−35^	4.83 × 10^−39^	4.98 × 10^−8^	0.566698	2.659583
	rank	1	3	2	1	8	7	4	10	6	5	9	11	12
F3	mean	0	3872.488	0	0	2.44 × 10^−12^	1.15 × 10^−10^	19362.44	15.49573	2.11 × 10^−14^	3.73 × 10^−24^	461.2824	376.5264	2104.13
	best	0	400.6281	0	0	6.00 × 10^−19^	1.33 × 10^−21^	2003.141	5.795644	2.29 × 10^−19^	2.13 × 10^−29^	238.6095	21.11739	1381.604
	worst	0	6730.25	0	0	1.39 × 10^−11^	1.89 × 10^−9^	33651.25	47.47647	3.92 × 10^−13^	3.50 × 10^−23^	1150.846	994.7339	3355.513
	std	0	1829.604	0	0	4.69 × 10^−12^	4.66 × 10^−10^	9148.022	11.50818	9.64 × 10^−14^	1.16 × 10^−23^	235.4943	308.3468	683.8623
	median	0	3943.313	0	0	1.77 × 10^−13^	1.04 × 10^−13^	19,716.57	11.52407	4.52 × 10^−16^	3.92 × 10^−26^	388.3647	284.2824	2037.889
	rank	1	10	1	1	4	5	11	6	3	2	8	7	9
F4	mean	0	10.05438	3.10 × 10^−265^	0	2.89 × 10^−19^	0.004291	50.27188	0.530759	1.19 × 10^−14^	1.78 × 10^−30^	1.198928	6.092114	2.744796
	best	0	0.175505	0	0	2.93 × 10^−20^	9.37 × 10^−5^	0.877525	0.257974	6.36 × 10^−16^	5.64 × 10^−32^	9.60 × 10^−9^	2.221789	2.150196
	worst	0	17.79352	4.40 × 10^−264^	0	9.32 × 10^−19^	0.034756	88.96761	0.934252	5.57 × 10^−14^	7.88 × 10^−30^	4.780357	12.96077	3.873355
	std	0	6.331918	0	0	2.45 × 10^−19^	0.008493	31.65959	0.20548	1.56 × 10^−14^	2.56 × 10^−30^	1.482929	2.675169	0.499179
	median	0	10.75345	1.90 × 10^−282^	0	2.51 × 10^−19^	0.001426	53.76726	0.515167	6.16 × 10^−15^	6.33 × 10^−31^	0.87983	5.706585	2.700252
	rank	1	11	2	1	4	6	12	7	5	3	8	10	9
F5	mean	0	10.34262	1.39 × 10^−5^	12.60997	22.6266	27.62587	26.49314	93.34453	25.7868	25.98698	42.73281	4474.037	577.5834
	best	0	5.208632	1.35 × 10^−6^	8.44 × 10^−29^	22.12664	24.90348	25.92307	26.80555	24.80211	24.82359	25.11065	25.49519	221.9666
	worst	0	16.82213	5.73 × 10^−5^	28.12341	23.3302	28.02781	27.87618	366.6048	26.34408	27.89297	162.2436	87,383.97	2189.572
	std	0	6.306277	1.55 × 10^−5^	15.76275	0.415468	0.84255	0.617609	108.4702	0.562673	1.000998	47.38394	21,505.67	454.3321
	median	0	5.493341	9.10 × 10^−6^	1.19 × 10^−28^	22.59841	27.96078	26.27693	29.12051	25.44735	25.54065	25.55866	83.52371	461.3534
	rank	1	3	2	4	5	9	8	11	6	7	10	13	12
F6	mean	0	2.521744	4.83 × 10^−8^	6.264793	1.75 × 10^−9^	3.571818	0.079134	0.146488	0.64109	1.223689	1.02 × 10^−16^	0.061549	33.12645
	best	0	1.427146	6.90 × 10^−9^	3.553726	7.84 × 10^−10^	2.476483	0.010206	0.076864	0.239352	0.226151	5.36 × 10^−17^	1.85 × 10^−6^	15.14563
	worst	0	2.876686	1.32 × 10^−7^	7.033228	4.66 × 10^−9^	4.644524	0.316978	0.242632	1.214835	2.100066	1.75 × 10^−16^	0.525533	60.89028
	std	0	0.435018	3.51 × 10^−8^	1.098922	1.00 × 10^−9^	0.741235	0.108637	0.050652	0.327781	0.531559	3.97 × 10^−17^	0.158821	14.48562
	median	0	2.674209	4.47 × 10^−8^	6.679094	1.55 × 10^−9^	3.682495	0.030662	0.155367	0.705569	1.181024	9.19 × 10^−17^	0.001996	30.73489
	rank	1	10	4	12	3	11	6	7	8	9	2	5	13
F7	mean	2.54 × 10^−5^	2.85 × 10^−4^	6.14 × 10^−5^	2.99 × 10^−5^	0.000531	0.004214	0.001241	0.011268	0.000807	0.001485	0.051231	0.178636	0.010273
	best	2.35 × 10^−6^	9.02 × 10^−6^	1.34 × 10^−6^	3.49 × 10^−6^	0.000108	0.001449	2.07 × 10^−5^	0.003853	0.000177	8.77 × 10^−5^	0.013704	0.066954	0.002942
	worst	6.89 × 10^−5^	0.001072	0.000256	0.000129	0.000873	0.009676	0.005238	0.021895	0.001899	0.002859	0.092717	0.399054	0.021284
	std	2.18 × 10^−5^	3.09 × 10^−4^	7.84 × 10^−5^	3.69 × 10^−5^	0.00023	0.002503	0.001545	0.005381	0.000499	0.00094	0.026681	0.084471	0.005152
	median	1.83 × 10^−5^	2.00 × 10^−4^	3.95 × 10^−5^	1.51 × 10^−5^	0.000518	0.003611	0.000794	0.010978	0.00082	0.001462	0.050284	0.172417	0.009875
	rank	1	4	3	2	5	9	7	11	6	8	12	13	10
Sum rank		7	43	15	22	35	54	51	62	39	38	57	68	76
Mean rank		1	6.1428571	2.1428571	3.1428571	5	7.7142857	7.2857143	8.8571429	5.5714286	5.4285714	8.1428571	9.7142857	10.857143
Total rank		1	7	2	3	4	9	8	11	6	5	10	12	13

**Table 3:** Optimization results for high-dimensional multimodal functions (F8–F13).

		DA	WSO	AVOA	RSA	MPA	TSA	WOA	MVO	GWO	TLBO	GSA	PSO	GA
F8	mean	−12,498.587	−9469.161	−12,471.545	−5647.3779	−9771.5065	−6329.3267	−11,107.96	−7972.4501	−6271.5652	−5804.7042	−3071.8049	−6725.3549	−8543.4031
	best	−12,622.812	−9866.2622	−12,571.081	−5864.3072	−10,538.388	−7476.6259	−12,569.905	−9290.7597	−7026.1104	−7194.9064	−4232.9513	−8358.1095	−9767.7805
	worst	−11,936.272	−8854.7341	−11,917.074	−5130.8349	−9190.6957	−4611.0881	−7883.2501	−7049.8979	−5274.2783	−4791.287	−2440.9757	−5216.7278	−7194.9171
	std	209.81989	395.93068	208.38669	240.75731	395.20999	781.55525	1854.4961	780.48164	511.88428	652.44489	532.29246	797.42343	685.87708
	median	−12,577.838	−9593.2117	−12,568.073	−5693.4379	−9800.0269	−6292.0143	−12,056.375	−7854.7623	−6267.6723	−5820.1694	−2989.8155	−6867.7852	−8524.8117
	rank	1	5	2	12	4	9	3	7	10	11	13	8	6
F9	mean	0	0	0	0	0	167.94776	0	94.904612	1.654 × 10^−14^	0	27.653242	65.689736	53.046261
	best	0	0	0	0	0	87.061474	0	51.208516	0	0	13.512937	38.60839	22.537741
	worst	0	0	0	0	0	279.5677	0	144.81711	1.103 × 10^−13^	0	47.295235	111.13666	74.601525
	std	0	0	0	0	0	54.529301	0	26.93684	3.471 × 10^−14^	0	9.7990172	20.142107	14.760996
	median	0	0	0	0	0	161.69195	0	94.18019	0	0	25.578047	63.123014	51.041263
	rank	1	1	1	1	1	7	1	6	2	1	3	5	4
F10	mean	8.88 × 10^−16^	1.509 × 10^−15^	8.88 × 10^−16^	8.88 × 10^−16^	4.16 × 10^−15^	1.2053421	3.99 × 10^−15^	0.5606201	1.62 × 10^−14^	4.33 × 10^−15^	7.97 × 10^−9^	2.6456892	3.4682044
	best	8.88 × 10^−16^	8.882 × 10^−16^	8.88 × 10^−16^	8.88 × 10^−16^	8.88 × 10^−16^	7.78 × 10^−15^	8.88 × 10^−16^	0.0975925	7.78 × 10^−15^	4.33 × 10^−15^	4.52 × 10^−9^	1.6428153	2.7957914
	worst	8.88 × 10^−16^	2.267 × 10^−15^	8.88 × 10^−16^	8.88 × 10^−16^	4.33 × 10^−15^	3.2725869	7.78 × 10^−15^	2.4399845	2.16 × 10^−14^	4.33 × 10^−15^	1.40 × 10^−8^	4.9058652	4.5031721
	std	0	4.867 × 10^−16^	0	0	8.493 × 10^−16^	1.6778805	2.43 × 10^−15^	0.7239449	3.79 × 10^−15^	8.92 × 10^−31^	2.50 × 10^−9^	0.9170296	0.4240318
	median	8.88 × 10^−16^	1.577 × 10^−15^	8.88 × 10^−16^	8.88 × 10^−16^	4.33 × 10^−15^	2.16 × 10^−14^	4.33 × 10^−15^	0.1885052	1.47 × 10^−14^	4.33 × 10^−15^	7.49 × 10^−9^	2.6521765	3.5210558
	rank	1	2	1	1	4	9	3	8	6	5	7	10	11
F11	mean	0	0	0	0	0	0.0085784	0	0.387725	0.0012996	0	6.9924949	0.1797264	1.4294144
	best	0	0	0	0	0	0	0	0.246549	0	0	2.906073	0.0022963	1.2495814
	worst	0	0	0	0	0	0.0199327	0	0.51996	0.0182608	0	12.259906	0.8496609	1.6742558
	std	0	0	0	0	0	0.0067277	0	0.0875091	0.0047931	0	2.9087854	0.244264	0.1324216
	median	0	0	0	0	0	0.0087247	0	0.4040637	0	0	7.0925272	0.1186979	1.4044224
	rank	1	1	1	1	1	3	1	5	2	1	7	4	6
F12	mean	1.57 × 10^−32^	0.5151867	2.50 × 10^−9^	1.2782189	1.98 × 10^−10^	5.619587	0.0194956	0.887294	0.0386861	0.0691962	0.2037572	1.4561765	0.2666751
	best	1.57 × 10^−32^	0.3011368	3.91 × 10^−10^	0.7461802	5.03 × 10^−11^	1.0058562	0.0011898	0.0009693	0.0121867	0.0233888	4.61 × 10^−19^	0.0001036	0.0590214
	worst	1.57 × 10^−32^	0.645715	7.60 × 10^−9^	1.5966928	3.70 × 10^−10^	13.713329	0.132808	3.7329882	0.0841886	0.1310947	0.9039107	5.0631656	0.6313819
	std	3.09 × 10^−48^	0.1309077	1.77 × 10^−9^	0.3248496	1.03 × 10^−10^	4.1483798	0.0427642	1.2793717	0.0228053	0.0223968	0.3286456	1.3743998	0.1482214
	median	1.57 × 10^−32^	0.5408114	2.32 × 10^−9^	1.347855	1.99 × 10^−10^	4.1761877	0.0056104	0.4077133	0.0367769	0.0666362	0.0778007	1.2468379	0.2565173
	rank	1	9	3	11	2	13	4	10	5	6	7	12	8
F13	mean	1.35 × 10^−32^	0.0416375	9.72 × 10^−9^	3.036 × 10^−31^	0.0024237	2.6356559	0.2081875	0.0317946	0.4984576	1.069047	0.0549666	3.4997534	2.6268705
	best	1.35 × 10^−32^	0.0072181	1.11 × 10^−9^	6.37 × 10^−32^	9.66 × 10^−10^	1.9522789	0.0360903	0.0062495	4.55 × 10^−5^	0.570896	4.52 × 10^−18^	0.0092863	1.2533292
	worst	1.35 × 10^−32^	0.1358809	3.69 × 10^−8^	5.275 × 10^−31^	0.0245565	3.6028899	0.6794044	0.0888872	0.9217114	1.4951226	0.9297198	12.20932	3.8224177
	std	3.09 × 10^−48^	0.0392387	9.38 × 10^−9^	2.406 × 10^−31^	0.0067815	0.5960279	0.1961933	0.0264985	0.2756329	0.247346	0.2284012	3.2403066	0.8065726
	median	1.35 × 10^−32^	0.0321681	6.33 × 10^−9^	3.89 × 10^−31^	2.74 × 10^−9^	2.4593686	0.1608405	0.022927	0.5016883	1.0812901	1.73 × 10^−17^	3.2069544	2.7814918
	rank	1	6	3	2	4	12	8	5	9	10	7	13	11
Sum rank		6	24	11	28	16	53	20	41	34	34	44	52	46
Mean rank		1	4	1.8333333	4.6666667	2.6666667	8.8333333	3.3333333	6.8333333	5.6666667	5.6666667	7.3333333	8.6666667	7.6666667
Total rank		1	5	2	6	3	12	4	8	7	7	9	11	10

**Table 4:** Optimization results for high-dimensional multimodal functions (F14–F23).

		DA	WSO	AVOA	RSA	MPA	TSA	WOA	MVO	GWO	TLBO	GSA	PSO	GA
F14	mean	0.998004	2.160282	1.094595	3.044554	1.009791	8.418526	2.523109	0.998356	3.614883	0.998357	3.485024	3.518472	1.047504
	best	0.998004	1.005785	0.998004	0.998035	0.998004	1.962309	0.998004	0.998004	0.998004	0.998004	0.998004	0.998004	0.998004
	worst	0.998004	5.912357	2.922781	12.3215	1.233486	15.07713	10.4712	1.005045	10.4712	1.005045	11.54481	12.3215	1.962315
	std	0.00 × 10^0^	1.501579	0.474205	3.267466	5.80 × 10^−2^	5.400611	3.149491	1.74 × 10^−3^	3.988315	0.001735	2.944019	4.050251	0.237313
	median	0.998004	1.767915	0.998004	2.188423	0.998004	11.39635	0.998007	0.998004	2.922781	0.998004	2.835084	1.96231	0.998006
	rank	1	7	6	9	4	13	8	2	12	3	10	11	5
F15	mean	0.000307	0.000767	0.000381	0.001125	0.001207	0.015971	0.000821	0.002604	0.003301	0.000612	0.002318	0.00246	0.014964
	best	0.000307	0.00053	0.00031	0.00074	0.000309	0.000311	0.000325	0.000311	0.000311	0.000316	0.000872	0.000308	0.000808
	worst	0.000307	0.00153	0.00072	0.002844	0.001674	0.107035	0.002235	0.019779	0.019805	0.001262	0.006801	0.019805	0.064966
	std	2.80 × 10^−19^	2.42 × 10^−4^	1.03 × 10^−4^	0.0005	0.000603	0.032098	0.000528	0.006477	0.00783	0.000428	0.001465	0.006552	0.017344
	median	0.000307	0.000713	0.00035	0.001025	0.0016	0.000866	0.000688	0.000691	0.000348	0.000358	0.002152	0.000348	0.013876
	rank	1	4	2	6	7	13	5	10	11	3	8	9	12
F16	mean	−1.03163	−1.0307	−1.03156	−1.02941	−1.02929	−1.03002	−1.03156	−1.03156	−1.03156	−1.03156	−1.03156	−1.03156	−1.03156
	best	−1.03163	−1.03162	−1.03163	−1.03161	−1.03163	−1.03163	−1.03163	−1.03163	−1.03163	−1.03163	−1.03163	−1.03163	−1.03163
	worst	−1.03163	−1.01936	−1.03071	−1.00095	−1.00093	−1.00092	−1.03071	−1.03071	−1.03071	−1.03071	−1.03071	−1.03071	−1.03071
	std	2.02 × 10^−16^	2.99 × 10^−3^	2.28 × 10^−4^	0.007468	7.61 × 10^−3^	0.007553	2.28 × 10^−4^	2.28 × 10^−4^	2.28 × 10^−4^	2.27 × 10^−4^	2.28 × 10^−4^	2.28 × 10^−4^	2.27 × 10^−4^
	median	−1.03163	−1.03144	−1.03163	−1.03129	−1.0316	−1.03163	−1.03163	−1.03163	−1.03163	−1.03163	−1.03163	−1.03163	−1.03163
	rank	1	9	3	11	12	10	4	6	5	8	3	2	7
F17	mean	0.397887	0.402833	0.397903	0.410229	0.398401	0.397939	0.397903	0.397903	0.397904	0.397973	0.397903	0.734284	0.464001
	best	0.397887	0.398225	0.397887	0.398605	0.397887	0.397891	0.397887	0.397887	0.397888	0.397895	0.397887	0.397887	0.397887
	worst	0.397887	0.431794	0.397985	0.482651	0.401154	0.398196	0.397986	0.397985	0.397986	0.398164	0.397985	2.719626	1.711687
	std	0	8.31 × 10^−3^	3.15 × 10^−5^	0.020788	0.001054	7.53 × 10^−5^	3.15 × 10^−5^	3.15 × 10^−5^	3.14 × 10^−5^	7.37 × 10^−5^	3.15 × 10^−5^	0.758272	0.323631
	median	0.397887	0.400179	0.39789	0.403606	0.397974	0.39791	0.39789	0.39789	0.39789	0.39797	0.39789	0.397899	0.397954
	rank	1	9	2	10	8	6	4	3	5	7	2	12	11
F18	mean	3	4.171269	3.094535	5.786357	6.161661	11.34212	3.094559	3.094534	3.094546	3.094535	3.094534	3.094534	7.268779
	best	3	3.00083	3.000417	3.001162	3.013933	3.000424	3.000417	3.000417	3.00042	3.000418	3.000417	3.000417	3.00124
	worst	3	13.9901	3.807338	30.47109	30.00128	89.37613	3.80734	3.807339	3.80735	3.807341	3.807338	3.807338	34.01482
	std	1.29 × 10^−15^	3.64 × 10^0^	2.10 × 10^−1^	9.094952	7.01 × 10^0^	27.97546	2.10 × 10^−1^	2.10 × 10^−1^	2.10 × 10^−1^	2.10 × 10^−1^	2.10 × 10^−1^	2.10 × 10^−1^	11.23307
	median	3	3.053139	3.016853	3.053149	3.563655	3.053143	3.016861	3.016853	3.016875	3.016854	3.016853	3.016853	3.06838
	rank	1	9	5	10	11	13	8	4	7	6	3	2	12
F19	mean	−3.86278	−3.84817	−3.85866	−3.83358	−3.72483	−3.85827	−3.85637	−3.85866	−3.85718	−3.85759	−3.85866	−3.85866	−3.8585
	best	−3.86278	−3.85883	−3.86278	−3.85506	−3.86278	−3.86267	−3.86276	−3.86278	−3.86278	−3.86262	−3.86278	−3.86278	−3.86276
	worst	−3.86278	−3.82608	−3.84575	−3.77745	−3.2931	−3.84566	−3.84532	−3.84575	−3.84563	−3.84549	−3.84575	−3.84575	−3.84542
	std	2.51 × 10^−15^	1.01 × 10^−2^	4.53 × 10^−3^	0.024329	1.51 × 10^−1^	4.53 × 10^−3^	0.004916	4.53 × 10^−3^	0.005091	0.00445	4.53 × 10^−3^	4.53 × 10^−3^	0.004685
	median	−3.86278	−3.84961	−3.85868	−3.83864	−3.72574	−3.85839	−3.8563	−3.85868	−3.8577	−3.85766	−3.85868	−3.85868	−3.85861
	rank	1	11	4	12	13	7	10	5	9	8	3	2	6
F20	mean	−3.322	−3.0476	−3.24649	−2.75829	−2.53258	−3.23345	−3.22845	−3.25216	−3.23731	−3.22151	−3.29839	−3.24273	−3.2075
	best	−3.322	−3.18611	−3.30805	−3.05537	−3.22483	−3.31688	−3.31358	−3.31909	−3.31909	−3.30209	−3.31909	−3.31909	−3.29395
	worst	−3.322	−2.5946	−3.16648	−1.67936	−1.78365	−3.07925	−3.07117	−3.16063	−3.05439	−2.98284	−3.276	−3.10298	−2.98977
	std	4.89 × 10^−16^	0.145291	0.065954	0.336712	0.37135	0.074112	0.089567	0.068096	0.085166	0.090058	1.11 × 10^−2^	0.084513	0.080321
	median	−3.322	−3.08055	−3.28035	−2.83178	−2.58954	−3.23325	−3.27796	−3.29507	−3.28035	−3.25796	−3.30009	−3.29107	−3.21953
	rank	1	11	4	12	13	7	8	3	6	9	2	5	10
F21	mean	−10.1532	−7.94843	−10.0756	−5.13005	−7.55876	−5.97405	−9.33081	−8.84583	−9.33558	−6.87384	−7.20503	−5.68167	−6.29906
	best	−10.1532	−8.17478	−10.1531	−5.20758	−10.1515	−10.127	−10.1524	−10.1531	−10.153	−9.39837	−10.1531	−10.1475	−9.68031
	worst	−10.1532	−7.0426	−10.0008	−5.0552	−5.0552	−2.68305	−5.06437	−5.05519	−5.09912	−3.38081	−2.75379	−2.71215	−2.46559
	std	2.29 × 10^−15^	0.405098	6.76 × 10^−2^	6.76 × 10^−2^	2.26 × 10^0^	3.469479	1.996729	2.400183	1.986866	2.217498	3.712456	3.07385	2.939109
	median	−10.1532	−8.08315	−10.0859	−5.14029	−7.90122	−5.05419	−10.059	−10.0256	−10.0613	−7.24723	−10.0008	−5.11971	−7.06191
	rank	1	6	2	13	7	11	4	5	3	9	8	12	10
F22	mean	−10.4029	−7.82607	−10.3338	−5.17743	−8.0897	−6.92044	−8.10796	−8.42438	−10.3333	−7.95399	−10.0683	−6.43396	−7.39333
	best	−10.4029	−8.33074	−10.4029	−5.24652	−10.4005	−10.3296	−10.3932	−10.3939	−10.4027	−9.96518	−10.4029	−10.3954	−9.9853
	worst	−10.4029	−6.53485	−10.244	−5.08767	−5.08767	−1.93014	−1.94988	−2.9428	−10.2426	−4.13942	−5.04174	−2.83532	−2.74891
	std	3.86 × 10^−15^	0.646553	6.90 × 10^−2^	6.90 × 10^−2^	2.31 × 10^0^	3.792798	3.253597	2.96704	0.069008	1.79517	1.31 × 10^0^	3.72882	2.073994
	median	−10.4029	−8.18093	−10.3624	−5.20602	−9.04577	−7.56887	−10.2407	−10.2624	−10.362	−8.30839	−10.3386	−5.15757	−7.84552
	rank	1	9	2	13	7	11	6	5	3	8	4	12	10
F23	mean	−10.5364	−8.01765	−10.4951	−5.24882	−9.15341	−7.467	−8.60049	−9.45263	−10.4946	−8.11805	−10.2535	−6.50253	−6.44368
	best	−10.5364	−8.4351	−10.5338	−5.28757	−10.4492	−10.4418	−10.5328	−10.5338	−10.5335	−9.70695	−10.5338	−10.5308	−10.0333
	worst	−10.5364	−6.67331	−10.3747	−5.12847	−5.12848	−2.65119	−1.89584	−5.17423	−10.3743	−4.40123	−5.66586	−2.60818	−2.60211
	std	3.05 × 10^−15^	0.713837	4.86 × 10^−2^	4.86 × 10^−2^	1.62 × 10^0^	3.695013	3.502551	2.367546	0.04855	1.77152	1.19 × 10^0^	4.112832	2.774049
	median	−10.5364	−8.39116	−10.5068	−5.26059	−9.54713	−10.2273	−10.4893	−10.4972	−10.5066	−8.6934	−10.5068	−4.00068	−6.9684
	rank	1	9	2	13	6	10	7	5	3	8	4	11	12
Sum rank		10	84	32	109	88	101	64	48	64	69	47	78	95
Mean rank		1	8.4	3.2	10.9	8.8	10.1	6.4	4.8	6.4	6.9	4.7	7.8	9.5
Total rank		1	8	2	12	9	11	5	4	5	6	3	7	10

**Table 5:** Optimization results for the CEC 2017 test suite.

		DA	WSO	AVOA	RSA	MPA	TSA	WOA	MVO	GWO	TLBO	GSA	PSO	GA
C17-F1	mean	1.00 × 10^2^	4.46 × 10^9^	3.73 × 10^3^	9.89 × 10^9^	3.42 × 10^7^	1.69 × 10^9^	6.25 × 10^6^	7.29 × 10^3^	8.55 × 10^7^	1.43 × 10^8^	7.26 × 10^2^	3.05 × 10^3^	1.15 × 10^7^
	best	1.00 × 10^2^	3.66 × 10^9^	1.15 × 10^2^	8.55 × 10^9^	1.09 × 10^4^	3.61 × 10^8^	4.55 × 10^6^	4.64 × 10^3^	2.69 × 10^4^	6.35 × 10^7^	1.00 × 10^2^	3.38 × 10^2^	5.95 × 10^6^
	worst	1.00 × 10^2^	5.82 × 10^9^	1.15 × 10^4^	1.18 × 10^+10^	1.24 × 10^8^	3.67 × 10^9^	8.23 × 10^6^	1.07 × 10^4^	3.10 × 10^8^	3.44 × 10^8^	1.74 × 10^3^	9.02 × 10^3^	1.65 × 10^7^
	std	0.00 × 10^0^	1.02 × 10^9^	5.81 × 10^3^	1.59 × 10^9^	6.56 × 10^7^	1.61 × 10^9^	1.69 × 10^6^	3.11 × 10^3^	1.64 × 10^8^	1.47 × 10^8^	7.71 × 10^2^	4.38 × 10^3^	4.79 × 10^6^
	median	1.00 × 10^2^	4.19 × 10^9^	1.62 × 10^3^	9.62 × 10^9^	6.27 × 10^6^	1.35 × 10^9^	6.11 × 10^6^	6.89 × 10^3^	1.57 × 10^7^	8.14 × 10^7^	5.34 × 10^2^	1.42 × 10^3^	1.17 × 10^7^
	rank	1	12	4	13	8	11	6	5	9	10	2	3	7
C17-F3	mean	3.00 × 10^2^	7.51 × 10^3^	3.02 × 10^2^	9.35 × 10^3^	1.37 × 10^3^	1.09 × 10^4^	1.68 × 10^3^	3.00 × 10^2^	2.98 × 10^3^	7.13 × 10^2^	9.94 × 10^3^	3.00 × 10^2^	1.43 × 10^4^
	best	3.00 × 10^2^	3.90 × 10^3^	3.00 × 10^2^	5.05 × 10^3^	7.76 × 10^2^	4.14 × 10^3^	6.09 × 10^2^	3.00 × 10^2^	1.49 × 10^3^	4.66 × 10^2^	6.26 × 10^3^	3.00 × 10^2^	4.22 × 10^3^
	worst	3.00 × 10^2^	1.02 × 10^4^	3.04 × 10^2^	1.25 × 10^4^	2.46 × 10^3^	1.53 × 10^4^	3.24 × 10^3^	3.00 × 10^2^	5.71 × 10^3^	8.74 × 10^2^	1.35 × 10^4^	3.00 × 10^2^	2.26 × 10^4^
	std	0.00 × 10^0^	2.94 × 10^3^	2.31 × 10^0^	3.71 × 10^3^	8.48 × 10^2^	5.18 × 10^3^	1.35 × 10^3^	5.17 × 10^−2^	2.12 × 10^3^	1.95 × 10^2^	3.25 × 10^3^	5.05 × 10^−14^	1.05 × 10^4^
	median	3.00 × 10^2^	7.95 × 10^3^	3.02 × 10^2^	9.93 × 10^3^	1.13 × 10^3^	1.20 × 10^4^	1.45 × 10^3^	3.00 × 10^2^	2.36 × 10^3^	7.56 × 10^2^	1.00 × 10^4^	3.00 × 10^2^	1.52 × 10^4^
	rank	1	9	4	10	6	12	7	3	8	5	11	2	13
C17-F4	mean	4.00 × 10^2^	8.27 × 10^2^	4.05 × 10^2^	1.32 × 10^3^	4.07 × 10^2^	5.71 × 10^2^	4.24 × 10^2^	4.03 × 10^2^	4.11 × 10^2^	4.09 × 10^2^	4.04 × 10^2^	4.20 × 10^2^	4.14 × 10^2^
	best	4.00 × 10^2^	6.44 × 10^2^	4.01 × 10^2^	8.31 × 10^2^	4.02 × 10^2^	4.75 × 10^2^	4.06 × 10^2^	4.02 × 10^2^	4.06 × 10^2^	4.08 × 10^2^	4.03 × 10^2^	4.00 × 10^2^	4.11 × 10^2^
	worst	4.00 × 10^2^	9.86 × 10^2^	4.06 × 10^2^	1.80 × 10^3^	4.11 × 10^2^	6.83 × 10^2^	4.71 × 10^2^	4.05 × 10^2^	4.27 × 10^2^	4.09 × 10^2^	4.06 × 10^2^	4.68 × 10^2^	4.18 × 10^2^
	std	0.00 × 10^0^	1.75 × 10^2^	2.63 × 10^0^	4.51 × 10^2^	4.65 × 10^0^	1.10 × 10^2^	3.41 × 10^1^	1.81 × 10^0^	1.17 × 10^1^	5.79 × 10^−1^	1.22 × 10^0^	3.56 × 10^1^	3.12 × 10^0^
	median	4.00 × 10^2^	8.40 × 10^2^	4.05 × 10^2^	1.33 × 10^3^	4.06 × 10^2^	5.63 × 10^2^	4.10 × 10^2^	4.03 × 10^2^	4.06 × 10^2^	4.09 × 10^2^	4.04 × 10^2^	4.05 × 10^2^	4.14 × 10^2^
	rank	1	12	4	13	5	11	10	2	7	6	3	9	8
C17-F5	mean	5.01 × 10^2^	5.59 × 10^2^	5.43 × 10^2^	5.71 × 10^2^	5.13 × 10^2^	5.63 × 10^2^	5.40 × 10^2^	5.23 × 10^2^	5.13 × 10^2^	5.33 × 10^2^	5.53 × 10^2^	5.27 × 10^2^	5.27 × 10^2^
	best	5.01 × 10^2^	5.45 × 10^2^	5.26 × 10^2^	5.57 × 10^2^	5.08 × 10^2^	5.42 × 10^2^	5.23 × 10^2^	5.10 × 10^2^	5.08 × 10^2^	5.28 × 10^2^	5.48 × 10^2^	5.11 × 10^2^	5.23 × 10^2^
	worst	5.02 × 10^2^	5.74 × 10^2^	5.62 × 10^2^	5.86 × 10^2^	5.18 × 10^2^	5.94 × 10^2^	5.75 × 10^2^	5.37 × 10^2^	5.20 × 10^2^	5.37 × 10^2^	5.64 × 10^2^	5.51 × 10^2^	5.33 × 10^2^
	std	5.41 × 10^−1^	1.36 × 10^1^	2.01 × 10^1^	1.75 × 10^1^	5.40 × 10^0^	2.51 × 10^1^	2.66 × 10^1^	1.24 × 10^1^	5.42 × 10^0^	4.22 × 10^0^	8.45 × 10^0^	2.00 × 10^1^	5.03 × 10^0^
	median	5.01 × 10^2^	5.60 × 10^2^	5.42 × 10^2^	5.71 × 10^2^	5.12 × 10^2^	5.58 × 10^2^	5.31 × 10^2^	5.23 × 10^2^	5.11 × 10^2^	5.34 × 10^2^	5.49 × 10^2^	5.24 × 10^2^	5.27 × 10^2^
	rank	1	11	9	13	2	12	8	4	3	7	10	5	6
C17-F6	mean	6.00 × 10^2^	6.30 × 10^2^	6.17 × 10^2^	6.40 × 10^2^	6.01 × 10^2^	6.24 × 10^2^	6.23 × 10^2^	6.02 × 10^2^	6.01 × 10^2^	6.07 × 10^2^	6.17 × 10^2^	6.07 × 10^2^	6.10 × 10^2^
	best	6.00 × 10^2^	6.24 × 10^2^	6.16 × 10^2^	6.37 × 10^2^	6.01 × 10^2^	6.15 × 10^2^	6.07 × 10^2^	6.00 × 10^2^	6.01 × 10^2^	6.05 × 10^2^	6.03 × 10^2^	6.01 × 10^2^	6.07 × 10^2^
	worst	6.00 × 10^2^	6.35 × 10^2^	6.20 × 10^2^	6.44 × 10^2^	6.02 × 10^2^	6.40 × 10^2^	6.44 × 10^2^	6.04 × 10^2^	6.02 × 10^2^	6.10 × 10^2^	6.36 × 10^2^	6.19 × 10^2^	6.14 × 10^2^
	std	0.00 × 10^0^	5.34 × 10^0^	1.82 × 10^0^	3.59 × 10^0^	8.61 × 10^−1^	1.17 × 10^1^	1.70 × 10^1^	1.85 × 10^0^	4.97 × 10^−1^	2.62 × 10^0^	1.64 × 10^1^	8.68 × 10^0^	3.60 × 10^0^
	median	6.00 × 10^2^	6.31 × 10^2^	6.16 × 10^2^	6.39 × 10^2^	6.01 × 10^2^	6.22 × 10^2^	6.20 × 10^2^	6.02 × 10^2^	6.01 × 10^2^	6.06 × 10^2^	6.15 × 10^2^	6.04 × 10^2^	6.10 × 10^2^
	rank	1	12	9	13	3	11	10	4	2	5	8	6	7
C17-F7	mean	7.11 × 10^2^	7.97 × 10^2^	7.65 × 10^2^	8.03 × 10^2^	7.24 × 10^2^	8.26 × 10^2^	7.61 × 10^2^	7.31 × 10^2^	7.26 × 10^2^	7.51 × 10^2^	7.17 × 10^2^	7.32 × 10^2^	7.36 × 10^2^
	best	7.11 × 10^2^	7.78 × 10^2^	7.43 × 10^2^	7.90 × 10^2^	7.20 × 10^2^	7.87 × 10^2^	7.50 × 10^2^	7.17 × 10^2^	7.17 × 10^2^	7.47 × 10^2^	7.15 × 10^2^	7.25 × 10^2^	7.26 × 10^2^
	worst	7.12 × 10^2^	8.11 × 10^2^	7.92 × 10^2^	8.15 × 10^2^	7.29 × 10^2^	8.67 × 10^2^	7.90 × 10^2^	7.49 × 10^2^	7.43 × 10^2^	7.59 × 10^2^	7.21 × 10^2^	7.44 × 10^2^	7.41 × 10^2^
	std	5.57 × 10^−1^	1.52 × 10^1^	2.43 × 10^1^	1.30 × 10^1^	3.88 × 10^0^	3.79 × 10^1^	2.11 × 10^1^	1.48 × 10^1^	1.28 × 10^1^	6.05 × 10^0^	2.78 × 10^0^	9.13 × 10^0^	7.49 × 10^0^
	median	7.11 × 10^2^	8.00 × 10^2^	7.62 × 10^2^	8.03 × 10^2^	7.24 × 10^2^	8.26 × 10^2^	7.52 × 10^2^	7.28 × 10^2^	7.21 × 10^2^	7.50 × 10^2^	7.16 × 10^2^	7.30 × 10^2^	7.39 × 10^2^
	rank	1	11	10	12	3	13	9	5	4	8	2	6	7
C17-F8	mean	8.01 × 10^2^	8.46 × 10^2^	8.31 × 10^2^	8.53 × 10^2^	8.12 × 10^2^	8.48 × 10^2^	8.36 × 10^2^	8.12 × 10^2^	8.16 × 10^2^	8.37 × 10^2^	8.20 × 10^2^	8.22 × 10^2^	8.17 × 10^2^
	best	8.01 × 10^2^	8.40 × 10^2^	8.20 × 10^2^	8.42 × 10^2^	8.09 × 10^2^	8.32 × 10^2^	8.18 × 10^2^	8.07 × 10^2^	8.10 × 10^2^	8.30 × 10^2^	8.12 × 10^2^	8.15 × 10^2^	8.13 × 10^2^
	worst	8.02 × 10^2^	8.54 × 10^2^	8.46 × 10^2^	8.58 × 10^2^	8.15 × 10^2^	8.66 × 10^2^	8.48 × 10^2^	8.16 × 10^2^	8.21 × 10^2^	8.45 × 10^2^	8.27 × 10^2^	8.29 × 10^2^	8.24 × 10^2^
	std	6.25 × 10^−1^	7.65 × 10^0^	1.20 × 10^1^	8.12 × 10^0^	2.95 × 10^0^	1.69 × 10^1^	1.38 × 10^1^	4.04 × 10^0^	4.62 × 10^0^	8.15 × 10^0^	7.11 × 10^0^	7.18 × 10^0^	5.66 × 10^0^
	median	8.01 × 10^2^	8.45 × 10^2^	8.28 × 10^2^	8.56 × 10^2^	8.13 × 10^2^	8.46 × 10^2^	8.39 × 10^2^	8.11 × 10^2^	8.16 × 10^2^	8.37 × 10^2^	8.20 × 10^2^	8.23 × 10^2^	8.15 × 10^2^
	rank	1	11	8	13	3	12	9	2	4	10	6	7	5
C17-F9	mean	9.00 × 10^2^	1.41 × 10^3^	1.18 × 10^3^	1.46 × 10^3^	9.05 × 10^2^	1.37 × 10^3^	1.37 × 10^3^	9.01 × 10^2^	9.12 × 10^2^	9.12 × 10^2^	9.00 × 10^2^	9.04 × 10^2^	9.05 × 10^2^
	best	9.00 × 10^2^	1.24 × 10^3^	9.53 × 10^2^	1.36 × 10^3^	9.00 × 10^2^	1.16 × 10^3^	1.07 × 10^3^	9.00 × 10^2^	9.01 × 10^2^	9.07 × 10^2^	9.00 × 10^2^	9.01 × 10^2^	9.03 × 10^2^
	worst	9.00 × 10^2^	1.57 × 10^3^	1.65 × 10^3^	1.59 × 10^3^	9.13 × 10^2^	1.66 × 10^3^	1.64 × 10^3^	9.03 × 10^2^	9.33 × 10^2^	9.20 × 10^2^	9.00 × 10^2^	9.12 × 10^2^	9.09 × 10^2^
	std	0.00 × 10^0^	1.54 × 10^2^	3.51 × 10^2^	1.06 × 10^2^	6.27 × 10^0^	2.32 × 10^2^	2.62 × 10^2^	1.65 × 10^0^	1.63 × 10^1^	6.01 × 10^0^	0.00 × 10^0^	5.83 × 10^0^	3.04 × 10^0^
	median	9.00 × 10^2^	1.41 × 10^3^	1.07 × 10^3^	1.44 × 10^3^	9.04 × 10^2^	1.34 × 10^3^	1.38 × 10^3^	9.00 × 10^2^	9.07 × 10^2^	9.10 × 10^2^	9.00 × 10^2^	9.02 × 10^2^	9.04 × 10^2^
	rank	1	11	8	12	5	10	9	2	7	6	1	3	4
C17-F10	mean	1.01 × 10^3^	2.22 × 10^3^	1.76 × 10^3^	2.54 × 10^3^	1.50 × 10^3^	2.01 × 10^3^	2.00 × 10^3^	1.76 × 10^3^	1.71 × 10^3^	2.14 × 10^3^	2.24 × 10^3^	1.92 × 10^3^	1.70 × 10^3^
	best	1.00 × 10^3^	1.92 × 10^3^	1.47 × 10^3^	2.37 × 10^3^	1.38 × 10^3^	1.74 × 10^3^	1.44 × 10^3^	1.44 × 10^3^	1.52 × 10^3^	1.76 × 10^3^	1.97 × 10^3^	1.55 × 10^3^	1.40 × 10^3^
	worst	1.01 × 10^3^	2.38 × 10^3^	2.38 × 10^3^	2.89 × 10^3^	1.58 × 10^3^	2.25 × 10^3^	2.51 × 10^3^	2.25 × 10^3^	1.97 × 10^3^	2.42 × 10^3^	2.35 × 10^3^	2.32 × 10^3^	2.08 × 10^3^
	std	7.24 × 10^0^	2.24 × 10^2^	4.63 × 10^2^	2.62 × 10^2^	9.99 × 10^1^	2.95 × 10^2^	5.64 × 10^2^	4.24 × 10^2^	2.04 × 10^2^	3.06 × 10^2^	1.98 × 10^2^	3.44 × 10^2^	3.16 × 10^2^
	median	1.01 × 10^3^	2.28 × 10^3^	1.59 × 10^3^	2.45 × 10^3^	1.53 × 10^3^	2.02 × 10^3^	2.02 × 10^3^	1.67 × 10^3^	1.67 × 10^3^	2.19 × 10^3^	2.33 × 10^3^	1.91 × 10^3^	1.65 × 10^3^
	rank	1	11	5	13	2	9	8	6	4	10	12	7	3
C17-F11	mean	1.10 × 10^3^	3.51 × 10^3^	1.15 × 10^3^	3.91 × 10^3^	1.13 × 10^3^	5.34 × 10^3^	1.15 × 10^3^	1.13 × 10^3^	1.15 × 10^3^	1.15 × 10^3^	1.14 × 10^3^	1.14 × 10^3^	2.35 × 10^3^
	best	1.10 × 10^3^	2.55 × 10^3^	1.12 × 10^3^	1.45 × 10^3^	1.11 × 10^3^	5.20 × 10^3^	1.11 × 10^3^	1.11 × 10^3^	1.12 × 10^3^	1.14 × 10^3^	1.12 × 10^3^	1.13 × 10^3^	1.11 × 10^3^
	worst	1.10 × 10^3^	4.44 × 10^3^	1.20 × 10^3^	6.33 × 10^3^	1.16 × 10^3^	5.42 × 10^3^	1.17 × 10^3^	1.15 × 10^3^	1.22 × 10^3^	1.17 × 10^3^	1.17 × 10^3^	1.16 × 10^3^	5.85 × 10^3^
	std	0.00 × 10^0^	9.23 × 10^2^	3.95 × 10^1^	2.39 × 10^3^	2.28 × 10^1^	1.08 × 10^2^	2.94 × 10^1^	2.29 × 10^1^	5.27 × 10^1^	1.58 × 10^1^	2.21 × 10^1^	1.56 × 10^1^	2.54 × 10^3^
	median	1.10 × 10^3^	3.53 × 10^3^	1.14 × 10^3^	3.92 × 10^3^	1.12 × 10^3^	5.37 × 10^3^	1.16 × 10^3^	1.13 × 10^3^	1.13 × 10^3^	1.15 × 10^3^	1.13 × 10^3^	1.14 × 10^3^	1.22 × 10^3^
	rank	1	11	6	12	2	13	8	3	9	7	4	5	10
C17-F12	mean	1.35 × 10^3^	2.76 × 10^8^	1.07 × 10^6^	6.88 × 10^8^	5.54 × 10^5^	1.01 × 10^6^	2.30 × 10^6^	1.00 × 10^6^	1.38 × 10^6^	4.93 × 10^6^	9.95 × 10^5^	7.92 × 10^3^	5.90 × 10^5^
	best	1.32 × 10^3^	6.24 × 10^7^	3.47 × 10^5^	1.53 × 10^8^	1.94 × 10^4^	5.26 × 10^5^	1.68 × 10^5^	8.65 × 10^3^	4.44 × 10^4^	1.32 × 10^6^	4.63 × 10^5^	2.49 × 10^3^	1.71 × 10^5^
	worst	1.44 × 10^3^	4.82 × 10^8^	1.95 × 10^6^	1.20 × 10^9^	8.67 × 10^5^	1.25 × 10^6^	3.81 × 10^6^	3.15 × 10^6^	2.16 × 10^6^	8.73 × 10^6^	1.68 × 10^6^	1.36 × 10^4^	1.04 × 10^6^
	std	6.23 × 10^1^	2.31 × 10^8^	8.14 × 10^5^	5.78 × 10^8^	4.06 × 10^5^	3.69 × 10^5^	1.84 × 10^6^	1.58 × 10^6^	1.02 × 10^6^	4.27 × 10^6^	5.62 × 10^5^	5.51 × 10^3^	3.89 × 10^5^
	median	1.33 × 10^3^	2.80 × 10^8^	1.00 × 10^6^	6.98 × 10^8^	6.64 × 10^5^	1.14 × 10^6^	2.60 × 10^6^	4.27 × 10^5^	1.66 × 10^6^	4.84 × 10^6^	9.18 × 10^5^	7.80 × 10^3^	5.74 × 10^5^
	rank	1	12	8	13	3	7	10	6	9	11	5	2	4
C17-F13	mean	1.31 × 10^3^	1.34 × 10^7^	1.79 × 10^4^	3.35 × 10^7^	5.33 × 10^3^	1.25 × 10^4^	7.42 × 10^3^	6.60 × 10^3^	1.01 × 10^4^	1.63 × 10^4^	9.86 × 10^3^	6.49 × 10^3^	5.31 × 10^4^
	best	1.30 × 10^3^	1.12 × 10^6^	2.69 × 10^3^	2.78 × 10^6^	3.66 × 10^3^	7.43 × 10^3^	3.23 × 10^3^	1.38 × 10^3^	6.38 × 10^3^	1.54 × 10^4^	4.96 × 10^3^	2.35 × 10^3^	8.37 × 10^3^
	worst	1.31 × 10^3^	4.45 × 10^7^	3.07 × 10^4^	1.11 × 10^8^	6.51 × 10^3^	1.97 × 10^4^	1.48 × 10^4^	1.21 × 10^4^	1.41 × 10^4^	1.86 × 10^4^	1.39 × 10^4^	1.63 × 10^4^	1.76 × 10^5^
	std	2.47 × 10^0^	2.26 × 10^7^	1.57 × 10^4^	5.65 × 10^7^	1.48 × 10^3^	5.77 × 10^3^	5.74 × 10^3^	6.04 × 10^3^	3.43 × 10^3^	1.63 × 10^3^	4.10 × 10^3^	7.22 × 10^3^	8.89 × 10^4^
	median	1.30 × 10^3^	4.01 × 10^6^	1.91 × 10^4^	1.00 × 10^7^	5.58 × 10^3^	1.13 × 10^4^	5.83 × 10^3^	6.44 × 10^3^	9.93 × 10^3^	1.57 × 10^4^	1.03 × 10^4^	3.64 × 10^3^	1.43 × 10^4^
	rank	1	12	10	13	2	8	5	4	7	9	6	3	11
C17-F14	mean	1.40 × 10^3^	3.56 × 10^3^	2.01 × 10^3^	5.25 × 10^3^	1.93 × 10^3^	3.34 × 10^3^	1.52 × 10^3^	1.57 × 10^3^	2.32 × 10^3^	1.59 × 10^3^	5.47 × 10^3^	2.96 × 10^3^	1.27 × 10^4^
	best	1.40 × 10^3^	2.79 × 10^3^	1.67 × 10^3^	4.60 × 10^3^	1.43 × 10^3^	1.49 × 10^3^	1.48 × 10^3^	1.42 × 10^3^	1.46 × 10^3^	1.51 × 10^3^	4.53 × 10^3^	1.43 × 10^3^	3.67 × 10^3^
	worst	1.40 × 10^3^	4.82 × 10^3^	2.79 × 10^3^	6.77 × 10^3^	2.87 × 10^3^	5.49 × 10^3^	1.56 × 10^3^	1.98 × 10^3^	4.88 × 10^3^	1.62 × 10^3^	7.41 × 10^3^	6.72 × 10^3^	2.53 × 10^4^
	std	5.41 × 10^−1^	1.03 × 10^3^	5.75 × 10^2^	1.11 × 10^3^	7.32 × 10^2^	2.31 × 10^3^	4.18 × 10^1^	2.99 × 10^2^	1.85 × 10^3^	5.32 × 10^1^	1.47 × 10^3^	2.75 × 10^3^	9.95 × 10^3^
	median	1.40 × 10^3^	3.31 × 10^3^	1.78 × 10^3^	4.82 × 10^3^	1.70 × 10^3^	3.19 × 10^3^	1.52 × 10^3^	1.43 × 10^3^	1.48 × 10^3^	1.61 × 10^3^	4.97 × 10^3^	1.84 × 10^3^	1.09 × 10^4^
	rank	1	10	6	11	5	9	2	3	7	4	12	8	13
C17-F15	mean	1.50 × 10^3^	9.33 × 10^3^	5.21 × 10^3^	1.36 × 10^4^	3.92 × 10^3^	6.87 × 10^3^	6.11 × 10^3^	1.54 × 10^3^	5.71 × 10^3^	1.70 × 10^3^	2.34 × 10^4^	8.82 × 10^3^	4.48 × 10^3^
	best	1.50 × 10^3^	3.16 × 10^3^	2.06 × 10^3^	2.71 × 10^3^	3.18 × 10^3^	2.30 × 10^3^	2.00 × 10^3^	1.53 × 10^3^	3.52 × 10^3^	1.58 × 10^3^	1.10 × 10^4^	2.84 × 10^3^	1.88 × 10^3^
	worst	1.50 × 10^3^	1.49 × 10^4^	1.24 × 10^4^	2.97 × 10^4^	4.81 × 10^3^	1.23 × 10^4^	1.32 × 10^4^	1.55 × 10^3^	6.77 × 10^3^	1.79 × 10^3^	3.50 × 10^4^	1.45 × 10^4^	7.86 × 10^3^
	std	2.56 × 10^−1^	5.40 × 10^3^	5.23 × 10^3^	1.28 × 10^4^	7.35 × 10^2^	4.67 × 10^3^	5.29 × 10^3^	1.30 × 10^1^	1.63 × 10^3^	1.12 × 10^2^	1.25 × 10^4^	5.29 × 10^3^	3.23 × 10^3^
	median	1.50 × 10^3^	9.64 × 10^3^	3.21 × 10^3^	1.10 × 10^4^	3.84 × 10^3^	6.45 × 10^3^	4.63 × 10^3^	1.54 × 10^3^	6.28 × 10^3^	1.72 × 10^3^	2.37 × 10^4^	8.98 × 10^3^	4.09 × 10^3^
	rank	1	11	6	12	4	9	8	2	7	3	13	10	5
C17-F16	mean	1.60 × 10^3^	2.00 × 10^3^	1.80 × 10^3^	2.01 × 10^3^	1.68 × 10^3^	2.04 × 10^3^	1.94 × 10^3^	1.81 × 10^3^	1.73 × 10^3^	1.68 × 10^3^	2.06 × 10^3^	1.92 × 10^3^	1.80 × 10^3^
	best	1.60 × 10^3^	1.93 × 10^3^	1.64 × 10^3^	1.81 × 10^3^	1.64 × 10^3^	1.86 × 10^3^	1.76 × 10^3^	1.72 × 10^3^	1.62 × 10^3^	1.65 × 10^3^	1.94 × 10^3^	1.82 × 10^3^	1.72 × 10^3^
	worst	1.60 × 10^3^	2.10 × 10^3^	1.92 × 10^3^	2.27 × 10^3^	1.71 × 10^3^	2.22 × 10^3^	2.07 × 10^3^	1.87 × 10^3^	1.82 × 10^3^	1.73 × 10^3^	2.25 × 10^3^	2.07 × 10^3^	1.83 × 10^3^
	std	3.44 × 10^−1^	8.17 × 10^1^	1.27 × 10^2^	2.11 × 10^2^	3.34 × 10^1^	1.78 × 10^2^	1.58 × 10^2^	6.80 × 10^1^	9.19 × 10^1^	3.97 × 10^1^	1.55 × 10^2^	1.28 × 10^2^	5.93 × 10^1^
	median	1.60 × 10^3^	1.98 × 10^3^	1.83 × 10^3^	1.97 × 10^3^	1.69 × 10^3^	2.04 × 10^3^	1.97 × 10^3^	1.82 × 10^3^	1.73 × 10^3^	1.66 × 10^3^	2.03 × 10^3^	1.89 × 10^3^	1.82 × 10^3^
	rank	1	10	6	11	3	12	9	7	4	2	13	8	5
C17-F17	mean	1.70 × 10^3^	1.82 × 10^3^	1.75 × 10^3^	1.82 × 10^3^	1.73 × 10^3^	1.80 × 10^3^	1.84 × 10^3^	1.84 × 10^3^	1.77 × 10^3^	1.76 × 10^3^	1.84 × 10^3^	1.75 × 10^3^	1.75 × 10^3^
	best	1.70 × 10^3^	1.80 × 10^3^	1.73 × 10^3^	1.80 × 10^3^	1.72 × 10^3^	1.78 × 10^3^	1.77 × 10^3^	1.78 × 10^3^	1.72 × 10^3^	1.75 × 10^3^	1.75 × 10^3^	1.74 × 10^3^	1.75 × 10^3^
	worst	1.70 × 10^3^	1.82 × 10^3^	1.79 × 10^3^	1.82 × 10^3^	1.77 × 10^3^	1.81 × 10^3^	1.88 × 10^3^	1.94 × 10^3^	1.87 × 10^3^	1.77 × 10^3^	1.97 × 10^3^	1.76 × 10^3^	1.76 × 10^3^
	std	1.69 × 10^−1^	1.16 × 10^1^	3.13 × 10^1^	1.23 × 10^1^	2.78 × 10^1^	1.19 × 10^1^	5.34 × 10^1^	8.65 × 10^1^	7.34 × 10^1^	1.06 × 10^1^	1.22 × 10^2^	6.06 × 10^0^	2.67 × 10^0^
	median	1.70 × 10^3^	1.82 × 10^3^	1.74 × 10^3^	1.82 × 10^3^	1.72 × 10^3^	1.80 × 10^3^	1.85 × 10^3^	1.82 × 10^3^	1.74 × 10^3^	1.76 × 10^3^	1.83 × 10^3^	1.75 × 10^3^	1.76 × 10^3^
	rank	1	10	3	9	2	8	11	12	7	6	13	4	5
C17-F18	mean	1.81 × 10^3^	2.23 × 10^6^	1.16 × 10^4^	5.55 × 10^6^	1.08 × 10^4^	1.18 × 10^4^	2.27 × 10^4^	2.04 × 10^4^	1.94 × 10^4^	2.88 × 10^4^	9.51 × 10^3^	2.14 × 10^4^	1.25 × 10^4^
	best	1.80 × 10^3^	1.17 × 10^5^	4.77 × 10^3^	2.75 × 10^5^	4.10 × 10^3^	7.32 × 10^3^	6.33 × 10^3^	8.52 × 10^3^	6.21 × 10^3^	2.34 × 10^4^	6.27 × 10^3^	2.85 × 10^3^	3.39 × 10^3^
	worst	1.82 × 10^3^	6.46 × 10^6^	1.52 × 10^4^	1.61 × 10^7^	1.61 × 10^4^	1.59 × 10^4^	3.57 × 10^4^	3.29 × 10^4^	3.28 × 10^4^	3.60 × 10^4^	1.16 × 10^4^	3.97 × 10^4^	1.80 × 10^4^
	std	1.09 × 10^1^	3.19 × 10^6^	5.11 × 10^3^	7.98 × 10^6^	5.96 × 10^3^	3.89 × 10^3^	1.54 × 10^4^	1.25 × 10^4^	1.46 × 10^4^	6.29 × 10^3^	2.47 × 10^3^	2.07 × 10^4^	6.96 × 10^3^
	median	1.80 × 10^3^	1.17 × 10^6^	1.32 × 10^4^	2.91 × 10^6^	1.15 × 10^4^	1.20 × 10^4^	2.45 × 10^4^	2.02 × 10^4^	1.94 × 10^4^	2.79 × 10^4^	1.01 × 10^4^	2.14 × 10^4^	1.43 × 10^4^
	rank	1	12	4	13	3	5	10	8	7	11	2	9	6
C17-F19	mean	1.90 × 10^3^	3.21 × 10^5^	6.58 × 10^3^	6.86 × 10^5^	5.50 × 10^3^	1.22 × 10^5^	3.39 × 10^4^	1.91 × 10^3^	5.29 × 10^3^	4.62 × 10^3^	3.94 × 10^4^	2.43 × 10^4^	6.07 × 10^3^
	best	1.90 × 10^3^	2.07 × 10^4^	2.17 × 10^3^	4.47 × 10^4^	2.31 × 10^3^	1.95 × 10^3^	7.51 × 10^3^	1.91 × 10^3^	1.94 × 10^3^	2.04 × 10^3^	1.09 × 10^4^	2.61 × 10^3^	2.21 × 10^3^
	worst	1.90 × 10^3^	6.72 × 10^5^	1.29 × 10^4^	1.47 × 10^6^	9.22 × 10^3^	2.44 × 10^5^	6.21 × 10^4^	1.92 × 10^3^	1.35 × 10^4^	1.22 × 10^4^	5.72 × 10^4^	7.49 × 10^4^	9.67 × 10^3^
	std	8.10 × 10^−1^	3.03 × 10^5^	5.70 × 10^3^	7.01 × 10^5^	3.83 × 10^3^	1.51 × 10^5^	2.44 × 10^4^	7.45 × 10^0^	6.01 × 10^3^	5.50 × 10^3^	2.26 × 10^4^	3.71 × 10^4^	3.35 × 10^3^
	median	1.90 × 10^3^	2.96 × 10^5^	5.61 × 10^3^	6.12 × 10^5^	5.24 × 10^3^	1.21 × 10^5^	3.31 × 10^4^	1.91 × 10^3^	2.87 × 10^3^	2.12 × 10^3^	4.48 × 10^4^	9.92 × 10^3^	6.20 × 10^3^
	rank	1	12	7	13	5	11	9	2	4	3	10	8	6
C17-F20	mean	2.00 × 10^3^	2.21 × 10^3^	2.17 × 10^3^	2.22 × 10^3^	2.09 × 10^3^	2.20 × 10^3^	2.20 × 10^3^	2.14 × 10^3^	2.17 × 10^3^	2.07 × 10^3^	2.25 × 10^3^	2.16 × 10^3^	2.05 × 10^3^
	best	2.00 × 10^3^	2.15 × 10^3^	2.03 × 10^3^	2.16 × 10^3^	2.07 × 10^3^	2.10 × 10^3^	2.10 × 10^3^	2.05 × 10^3^	2.13 × 10^3^	2.06 × 10^3^	2.18 × 10^3^	2.14 × 10^3^	2.03 × 10^3^
	worst	2.00 × 10^3^	2.28 × 10^3^	2.29 × 10^3^	2.27 × 10^3^	2.12 × 10^3^	2.31 × 10^3^	2.28 × 10^3^	2.24 × 10^3^	2.24 × 10^3^	2.08 × 10^3^	2.34 × 10^3^	2.20 × 10^3^	2.06 × 10^3^
	std	0.00 × 10^0^	5.88 × 10^1^	1.25 × 10^2^	5.94 × 10^1^	2.27 × 10^1^	9.61 × 10^1^	9.60 × 10^1^	8.72 × 10^1^	5.50 × 10^1^	9.53 × 10^0^	8.20 × 10^1^	2.95 × 10^1^	1.08 × 10^1^
	median	2.00 × 10^3^	2.20 × 10^3^	2.17 × 10^3^	2.22 × 10^3^	2.08 × 10^3^	2.20 × 10^3^	2.21 × 10^3^	2.13 × 10^3^	2.15 × 10^3^	2.07 × 10^3^	2.23 × 10^3^	2.16 × 10^3^	2.05 × 10^3^
	rank	1	11	8	12	4	10	9	5	7	3	13	6	2
C17-F21	mean	2.20 × 10^3^	2.29 × 10^3^	2.21 × 10^3^	2.27 × 10^3^	2.26 × 10^3^	2.32 × 10^3^	2.31 × 10^3^	2.25 × 10^3^	2.31 × 10^3^	2.30 × 10^3^	2.36 × 10^3^	2.32 × 10^3^	2.30 × 10^3^
	best	2.20 × 10^3^	2.24 × 10^3^	2.20 × 10^3^	2.22 × 10^3^	2.25 × 10^3^	2.22 × 10^3^	2.22 × 10^3^	2.20 × 10^3^	2.31 × 10^3^	2.20 × 10^3^	2.35 × 10^3^	2.31 × 10^3^	2.23 × 10^3^
	worst	2.20 × 10^3^	2.32 × 10^3^	2.24 × 10^3^	2.29 × 10^3^	2.26 × 10^3^	2.37 × 10^3^	2.35 × 10^3^	2.30 × 10^3^	2.32 × 10^3^	2.33 × 10^3^	2.38 × 10^3^	2.32 × 10^3^	2.33 × 10^3^
	std	0.00 × 10^0^	4.19 × 10^1^	1.79 × 10^1^	3.18 × 10^1^	2.26 × 10^0^	7.47 × 10^1^	6.55 × 10^1^	6.51 × 10^1^	4.00 × 10^0^	6.83 × 10^1^	1.54 × 10^1^	8.14 × 10^0^	5.13 × 10^1^
	median	2.20 × 10^3^	2.31 × 10^3^	2.21 × 10^3^	2.27 × 10^3^	2.26 × 10^3^	2.35 × 10^3^	2.33 × 10^3^	2.25 × 10^3^	2.31 × 10^3^	2.33 × 10^3^	2.36 × 10^3^	2.32 × 10^3^	2.31 × 10^3^
	rank	1	6	2	5	4	12	9	3	10	8	13	11	7
C17-F22	mean	2.30 × 10^3^	2.67 × 10^3^	2.31 × 10^3^	2.90 × 10^3^	2.30 × 10^3^	2.70 × 10^3^	2.32 × 10^3^	2.29 × 10^3^	2.31 × 10^3^	2.32 × 10^3^	2.30 × 10^3^	2.31 × 10^3^	2.32 × 10^3^
	best	2.30 × 10^3^	2.57 × 10^3^	2.30 × 10^3^	2.70 × 10^3^	2.30 × 10^3^	2.45 × 10^3^	2.32 × 10^3^	2.23 × 10^3^	2.30 × 10^3^	2.31 × 10^3^	2.30 × 10^3^	2.30 × 10^3^	2.31 × 10^3^
	worst	2.30 × 10^3^	2.79 × 10^3^	2.31 × 10^3^	3.05 × 10^3^	2.31 × 10^3^	2.91 × 10^3^	2.33 × 10^3^	2.31 × 10^3^	2.32 × 10^3^	2.33 × 10^3^	2.30 × 10^3^	2.34 × 10^3^	2.32 × 10^3^
	std	1.58 × 10^−1^	1.15 × 10^2^	3.31 × 10^0^	1.62 × 10^2^	3.76 × 10^0^	2.24 × 10^2^	5.83 × 10^0^	3.99 × 10^1^	1.03 × 10^1^	8.74 × 10^0^	1.45 × 10^−2^	2.28 × 10^1^	3.34 × 10^0^
	median	2.30 × 10^3^	2.66 × 10^3^	2.31 × 10^3^	2.93 × 10^3^	2.30 × 10^3^	2.73 × 10^3^	2.32 × 10^3^	2.30 × 10^3^	2.31 × 10^3^	2.32 × 10^3^	2.30 × 10^3^	2.30 × 10^3^	2.32 × 10^3^
	rank	3	11	6	13	4	12	10	1	5	9	2	7	8
C17-F23	mean	2.60 × 10^3^	2.69 × 10^3^	2.64 × 10^3^	2.70 × 10^3^	2.61 × 10^3^	2.72 × 10^3^	2.65 × 10^3^	2.62 × 10^3^	2.61 × 10^3^	2.64 × 10^3^	2.79 × 10^3^	2.64 × 10^3^	2.65 × 10^3^
	best	2.60 × 10^3^	2.65 × 10^3^	2.63 × 10^3^	2.67 × 10^3^	2.61 × 10^3^	2.63 × 10^3^	2.63 × 10^3^	2.61 × 10^3^	2.61 × 10^3^	2.63 × 10^3^	2.72 × 10^3^	2.64 × 10^3^	2.64 × 10^3^
	worst	2.60 × 10^3^	2.71 × 10^3^	2.66 × 10^3^	2.74 × 10^3^	2.62 × 10^3^	2.76 × 10^3^	2.67 × 10^3^	2.63 × 10^3^	2.62 × 10^3^	2.65 × 10^3^	2.92 × 10^3^	2.65 × 10^3^	2.66 × 10^3^
	std	1.44 × 10^0^	3.10 × 10^1^	1.47 × 10^1^	3.46 × 10^1^	2.57 × 10^0^	6.41 × 10^1^	2.19 × 10^1^	1.14 × 10^1^	6.91 × 10^0^	9.54 × 10^0^	1.02 × 10^2^	9.18 × 10^0^	1.44 × 10^1^
	median	2.60 × 10^3^	2.70 × 10^3^	2.64 × 10^3^	2.69 × 10^3^	2.61 × 10^3^	2.74 × 10^3^	2.65 × 10^3^	2.62 × 10^3^	2.61 × 10^3^	2.64 × 10^3^	2.75 × 10^3^	2.64 × 10^3^	2.66 × 10^3^
	rank	1	10	5	11	3	12	8	4	2	6	13	7	9
C17-F24	mean	2.63 × 10^3^	2.77 × 10^3^	2.76 × 10^3^	2.84 × 10^3^	2.63 × 10^3^	2.67 × 10^3^	2.76 × 10^3^	2.68 × 10^3^	2.75 × 10^3^	2.75 × 10^3^	2.74 × 10^3^	2.76 × 10^3^	2.72 × 10^3^
	best	2.52 × 10^3^	2.71 × 10^3^	2.73 × 10^3^	2.82 × 10^3^	2.61 × 10^3^	2.53 × 10^3^	2.73 × 10^3^	2.50 × 10^3^	2.72 × 10^3^	2.74 × 10^3^	2.50 × 10^3^	2.75 × 10^3^	2.54 × 10^3^
	worst	2.73 × 10^3^	2.84 × 10^3^	2.78 × 10^3^	2.91 × 10^3^	2.64 × 10^3^	2.81 × 10^3^	2.79 × 10^3^	2.76 × 10^3^	2.76 × 10^3^	2.77 × 10^3^	2.89 × 10^3^	2.79 × 10^3^	2.81 × 10^3^
	std	1.27 × 10^2^	6.89 × 10^1^	2.77 × 10^1^	4.55 × 10^1^	1.25 × 10^1^	1.63 × 10^2^	2.74 × 10^1^	1.32 × 10^2^	2.03 × 10^1^	1.43 × 10^1^	1.83 × 10^2^	1.65 × 10^1^	1.31 × 10^2^
	median	2.64 × 10^3^	2.75 × 10^3^	2.77 × 10^3^	2.82 × 10^3^	2.63 × 10^3^	2.66 × 10^3^	2.76 × 10^3^	2.73 × 10^3^	2.75 × 10^3^	2.75 × 10^3^	2.79 × 10^3^	2.76 × 10^3^	2.77 × 10^3^
	rank	1	12	11	13	2	3	9	4	7	8	6	10	5
C17-F25	mean	2.93 × 10^3^	3.12 × 10^3^	2.91 × 10^3^	3.27 × 10^3^	2.92 × 10^3^	3.13 × 10^3^	2.91 × 10^3^	2.92 × 10^3^	2.94 × 10^3^	2.93 × 10^3^	2.92 × 10^3^	2.92 × 10^3^	2.95 × 10^3^
	best	2.90 × 10^3^	3.04 × 10^3^	2.90 × 10^3^	3.20 × 10^3^	2.91 × 10^3^	2.91 × 10^3^	2.77 × 10^3^	2.90 × 10^3^	2.92 × 10^3^	2.92 × 10^3^	2.90 × 10^3^	2.90 × 10^3^	2.94 × 10^3^
	worst	2.95 × 10^3^	3.32 × 10^3^	2.95 × 10^3^	3.34 × 10^3^	2.92 × 10^3^	3.64 × 10^3^	2.96 × 10^3^	2.94 × 10^3^	2.95 × 10^3^	2.95 × 10^3^	2.94 × 10^3^	2.95 × 10^3^	2.96 × 10^3^
	std	2.51 × 10^1^	1.44 × 10^2^	2.55 × 10^1^	6.30 × 10^1^	4.53 × 10^0^	3.75 × 10^2^	1.01 × 10^2^	2.55 × 10^1^	1.22 × 10^1^	2.17 × 10^1^	2.37 × 10^1^	2.83 × 10^1^	1.17 × 10^1^
	median	2.94 × 10^3^	3.06 × 10^3^	2.90 × 10^3^	3.27 × 10^3^	2.92 × 10^3^	2.98 × 10^3^	2.95 × 10^3^	2.92 × 10^3^	2.94 × 10^3^	2.93 × 10^3^	2.92 × 10^3^	2.92 × 10^3^	2.95 × 10^3^
	rank	7	11	2	13	3	12	1	4	9	8	5	6	10
C17-F26	mean	2.90 × 10^3^	3.53 × 10^3^	2.98 × 10^3^	3.74 × 10^3^	3.01 × 10^3^	3.60 × 10^3^	3.18 × 10^3^	2.90 × 10^3^	3.26 × 10^3^	3.20 × 10^3^	3.84 × 10^3^	2.90 × 10^3^	2.90 × 10^3^
	best	2.90 × 10^3^	3.20 × 10^3^	2.81 × 10^3^	3.42 × 10^3^	2.89 × 10^3^	3.14 × 10^3^	2.93 × 10^3^	2.90 × 10^3^	2.97 × 10^3^	2.91 × 10^3^	2.81 × 10^3^	2.81 × 10^3^	2.71 × 10^3^
	worst	2.90 × 10^3^	3.73 × 10^3^	3.15 × 10^3^	4.07 × 10^3^	3.28 × 10^3^	4.24 × 10^3^	3.58 × 10^3^	2.90 × 10^3^	3.88 × 10^3^	3.85 × 10^3^	4.32 × 10^3^	3.01 × 10^3^	3.10 × 10^3^
	std	4.04 × 10^−13^	2.74 × 10^2^	2.12 × 10^2^	3.03 × 10^2^	2.01 × 10^2^	5.85 × 10^2^	3.10 × 10^2^	3.80 × 10^−2^	4.59 × 10^2^	4.77 × 10^2^	7.59 × 10^2^	8.79 × 10^1^	2.16 × 10^2^
	median	2.90 × 10^3^	3.59 × 10^3^	2.98 × 10^3^	3.73 × 10^3^	2.93 × 10^3^	3.52 × 10^3^	3.10 × 10^3^	2.90 × 10^3^	3.09 × 10^3^	3.02 × 10^3^	4.12 × 10^3^	2.90 × 10^3^	2.89 × 10^3^
	rank	2	10	5	12	6	11	7	3	9	8	13	4	1
C17-F27	mean	3.09 × 10^3^	3.20 × 10^3^	3.12 × 10^3^	3.23 × 10^3^	3.10 × 10^3^	3.18 × 10^3^	3.19 × 10^3^	3.09 × 10^3^	3.12 × 10^3^	3.11 × 10^3^	3.22 × 10^3^	3.13 × 10^3^	3.16 × 10^3^
	best	3.09 × 10^3^	3.17 × 10^3^	3.10 × 10^3^	3.13 × 10^3^	3.09 × 10^3^	3.10 × 10^3^	3.18 × 10^3^	3.09 × 10^3^	3.09 × 10^3^	3.10 × 10^3^	3.21 × 10^3^	3.10 × 10^3^	3.12 × 10^3^
	worst	3.09 × 10^3^	3.26 × 10^3^	3.18 × 10^3^	3.42 × 10^3^	3.13 × 10^3^	3.22 × 10^3^	3.20 × 10^3^	3.09 × 10^3^	3.17 × 10^3^	3.17 × 10^3^	3.24 × 10^3^	3.18 × 10^3^	3.22 × 10^3^
	std	2.86 × 10^−13^	4.12 × 10^1^	4.33 × 10^1^	1.39 × 10^2^	2.08 × 10^1^	5.75 × 10^1^	1.23 × 10^1^	2.63 × 10^0^	4.30 × 10^1^	3.98 × 10^1^	1.59 × 10^1^	3.86 × 10^1^	4.47 × 10^1^
	median	3.09 × 10^3^	3.19 × 10^3^	3.10 × 10^3^	3.18 × 10^3^	3.10 × 10^3^	3.19 × 10^3^	3.19 × 10^3^	3.09 × 10^3^	3.10 × 10^3^	3.10 × 10^3^	3.22 × 10^3^	3.13 × 10^3^	3.15 × 10^3^
	rank	1	11	6	13	3	9	10	2	5	4	12	7	8
C17-F28	mean	3.10 × 10^3^	3.56 × 10^3^	3.23 × 10^3^	3.76 × 10^3^	3.22 × 10^3^	3.57 × 10^3^	3.28 × 10^3^	3.24 × 10^3^	3.34 × 10^3^	3.32 × 10^3^	3.44 × 10^3^	3.30 × 10^3^	3.24 × 10^3^
	best	3.10 × 10^3^	3.50 × 10^3^	3.10 × 10^3^	3.68 × 10^3^	3.17 × 10^3^	3.40 × 10^3^	3.15 × 10^3^	3.10 × 10^3^	3.19 × 10^3^	3.21 × 10^3^	3.43 × 10^3^	3.18 × 10^3^	3.14 × 10^3^
	worst	3.10 × 10^3^	3.62 × 10^3^	3.38 × 10^3^	3.82 × 10^3^	3.24 × 10^3^	3.78 × 10^3^	3.38 × 10^3^	3.38 × 10^3^	3.40 × 10^3^	3.38 × 10^3^	3.46 × 10^3^	3.38 × 10^3^	3.50 × 10^3^
	std	0.00 × 10^0^	5.65 × 10^1^	1.36 × 10^2^	6.98 × 10^1^	3.76 × 10^1^	2.11 × 10^2^	1.30 × 10^2^	1.70 × 10^2^	1.07 × 10^2^	8.95 × 10^1^	1.56 × 10^1^	1.03 × 10^2^	1.90 × 10^2^
	median	3.10 × 10^3^	3.57 × 10^3^	3.22 × 10^3^	3.77 × 10^3^	3.23 × 10^3^	3.56 × 10^3^	3.30 × 10^3^	3.23 × 10^3^	3.38 × 10^3^	3.34 × 10^3^	3.44 × 10^3^	3.32 × 10^3^	3.16 × 10^3^
	rank	1	11	3	13	2	12	6	4	9	8	10	7	5
C17-F29	mean	3.13 × 10^3^	3.32 × 10^3^	3.28 × 10^3^	3.37 × 10^3^	3.20 × 10^3^	3.23 × 10^3^	3.34 × 10^3^	3.20 × 10^3^	3.26 × 10^3^	3.21 × 10^3^	3.34 × 10^3^	3.26 × 10^3^	3.23 × 10^3^
	best	3.13 × 10^3^	3.31 × 10^3^	3.21 × 10^3^	3.30 × 10^3^	3.17 × 10^3^	3.17 × 10^3^	3.23 × 10^3^	3.14 × 10^3^	3.19 × 10^3^	3.16 × 10^3^	3.23 × 10^3^	3.17 × 10^3^	3.19 × 10^3^
	worst	3.13 × 10^3^	3.33 × 10^3^	3.36 × 10^3^	3.44 × 10^3^	3.24 × 10^3^	3.30 × 10^3^	3.49 × 10^3^	3.28 × 10^3^	3.37 × 10^3^	3.23 × 10^3^	3.62 × 10^3^	3.34 × 10^3^	3.28 × 10^3^
	std	2.70 × 10^0^	8.34 × 10^0^	8.49 × 10^1^	7.59 × 10^1^	3.68 × 10^1^	6.09 × 10^1^	1.16 × 10^2^	6.48 × 10^1^	9.58 × 10^1^	3.48 × 10^1^	2.06 × 10^2^	8.74 × 10^1^	4.37 × 10^1^
	median	3.13 × 10^3^	3.32 × 10^3^	3.28 × 10^3^	3.37 × 10^3^	3.20 × 10^3^	3.23 × 10^3^	3.33 × 10^3^	3.19 × 10^3^	3.24 × 10^3^	3.22 × 10^3^	3.25 × 10^3^	3.27 × 10^3^	3.23 × 10^3^
	rank	1	10	9	13	3	5	12	2	7	4	11	8	6
C17-F30	mean	3.42 × 10^3^	1.90 × 10^6^	2.87 × 10^5^	3.57 × 10^6^	4.03 × 10^5^	5.98 × 10^5^	9.65 × 10^5^	2.95 × 10^5^	9.10 × 10^5^	5.91 × 10^4^	7.61 × 10^5^	3.77 × 10^5^	1.49 × 10^6^
	best	3.39 × 10^3^	1.59 × 10^6^	1.02 × 10^5^	8.05 × 10^5^	1.56 × 10^4^	1.09 × 10^5^	4.44 × 10^3^	7.33 × 10^3^	3.28 × 10^4^	2.86 × 10^4^	5.85 × 10^5^	6.31 × 10^3^	5.11 × 10^5^
	worst	3.44 × 10^3^	2.70 × 10^6^	7.47 × 10^5^	5.65 × 10^6^	5.95 × 10^5^	1.26 × 10^6^	3.64 × 10^6^	1.12 × 10^6^	1.32 × 10^6^	9.91 × 10^4^	9.72 × 10^5^	7.47 × 10^5^	3.38 × 10^6^
	std	3.02 × 10^1^	5.82 × 10^5^	3.35 × 10^5^	2.21 × 10^6^	2.86 × 10^5^	5.34 × 10^5^	1.94 × 10^6^	6.01 × 10^5^	6.57 × 10^5^	3.74 × 10^4^	1.75 × 10^5^	4.64 × 10^5^	1.47 × 10^6^
	median	3.42 × 10^3^	1.65 × 10^6^	1.49 × 10^5^	3.92 × 10^6^	5.01 × 10^5^	5.09 × 10^5^	1.06 × 10^5^	2.41 × 10^4^	1.15 × 10^6^	5.43 × 10^4^	7.44 × 10^5^	3.77 × 10^5^	1.02 × 10^6^
	rank	1	12	3	13	6	7	10	4	9	2	8	5	11
Sum rank		38	315	177	350	106	288	239	116	188	191	239	183	197
Mean rank		1.31 × 10^0^	1.09 × 10^1^	6.10 × 10^0^	1.21 × 10^1^	3.66 × 10^0^	9.93 × 10^0^	8.24 × 10^0^	4.00 × 10^0^	6.48 × 10^0^	6.59 × 10^0^	8.24 × 10^0^	6.31 × 10^0^	6.79 × 10^0^
Total rank		1	11	4	12	2	10	9	3	6	7	9	5	8

**Table 6:** Wilcoxon signed-rank test results.

Compared Algorithm	Objective Function Type			
	Unimodal	High-Dimensional	Fixed-Dimensional	CEC 2017
DA vs. WSO	1.03 × 10^−24^	1.87 × 10^−21^	1.07 × 10^−4^	1.63 × 10^−19^
DA vs. AVOA	5.49 × 10^−2^	1.62 × 10^−5^	1.21 × 10^−18^	1.87 × 10^−21^
DA vs. RSA	1.05 × 10^−5^	4.89 × 10^−11^	1.37 × 10^−34^	1.87 × 10^−21^
DA vs. MPA	9.60 × 10^−25^	6.63 × 10^−15^	9.69 × 10^−9^	1.16 × 10^−18^
DA vs. TSA	9.60 × 10^−25^	1.22 × 10^−19^	1.37 × 10^−34^	2.29 × 10^−21^
DA vs. WOA	9.60 × 10^−25^	4.90 × 10^−14^	1.37 × 10^−34^	5.63 × 10^−21^
DA vs. MVO	9.60 × 10^−25^	1.87 × 10^−21^	1.37 × 10^−34^	1.10 × 10^−20^
DA vs. GWO	9.60 × 10^−25^	7.20 × 10^−16^	1.37 × 10^−34^	1.87 × 10^−21^
DA vs. TLBO	9.60 × 10^−25^	9.88 × 10^−15^	1.37 × 10^−34^	6.70 × 10^−21^
DA vs. GSA	9.60 × 10^−25^	1.87 × 10^−21^	1.39 × 10^−13^	2.02 × 10^−21^
DA vs. PSO	9.60 × 10^−25^	1.87 × 10^−21^	1.14 × 10^−16^	1.87 × 10^−21^
DA vs. GA	9.60 × 10^−25^	1.87 × 10^−21^	1.37 × 10^−34^	1.99 × 10^−20^

**Table 7:** Optimization results for the CEC 2011 test suite.

		DA	WSO	AVOA	RSA	MPA	TSA	WOA	MVO	GWO	TLBO	GSA	PSO	GA
C11-F1	mean	5.92 × 10^0^	1.80 × 10^1^	1.31 × 10^1^	2.23 × 10^1^	7.61 × 10^0^	1.87 × 10^1^	1.34 × 10^1^	1.42 × 10^1^	1.10 × 10^1^	1.87 × 10^1^	2.21 × 10^1^	1.82 × 10^1^	2.38 × 10^1^
	best	2.00 × 10^−10^	1.57 × 10^1^	9.04 × 10^0^	2.06 × 10^1^	3.79 × 10^−1^	1.79 × 10^1^	8.40 × 10^0^	1.17 × 10^1^	1.14 × 10^0^	1.72 × 10^1^	2.00 × 10^1^	1.07 × 10^1^	2.27 × 10^1^
	worst	1.23 × 10^1^	2.07 × 10^1^	1.70 × 10^1^	2.48 × 10^1^	1.27 × 10^1^	2.01 × 10^1^	1.75 × 10^1^	1.64 × 10^1^	1.78 × 10^1^	2.04 × 10^1^	2.35 × 10^1^	2.46 × 10^1^	2.59 × 10^1^
	std	7.20 × 10^0^	2.62 × 10^0^	4.64 × 10^0^	2.15 × 10^0^	5.93 × 10^0^	1.06 × 10^0^	4.40 × 10^0^	2.40 × 10^0^	7.42 × 10^0^	1.38 × 10^0^	1.55 × 10^0^	6.80 × 10^0^	1.50 × 10^0^
	median	5.69 × 10^0^	1.77 × 10^1^	1.32 × 10^1^	2.20 × 10^1^	8.68 × 10^0^	1.84 × 10^1^	1.39 × 10^1^	1.44 × 10^1^	1.25 × 10^1^	1.87 × 10^1^	2.23 × 10^1^	1.89 × 10^1^	2.33 × 10^1^
	rank	1	7	4	12	2	9	5	6	3	10	11	8	13
C11-F2	mean	−2.63 × 10^1^	−1.42 × 10^1^	−2.10 × 10^1^	−1.14 × 10^1^	−2.51 × 10^1^	−1.11 × 10^1^	−1.85 × 10^1^	−8.59 × 10^0^	−2.26 × 10^1^	−1.07 × 10^1^	−1.54 × 10^1^	−2.26 × 10^1^	−1.28 × 10^1^
	best	−2.71 × 10^1^	−1.56 × 10^1^	−2.15 × 10^1^	−1.18 × 10^1^	−2.57 × 10^1^	−1.49 × 10^1^	−2.20 × 10^1^	−1.06 × 10^1^	−2.47 × 10^1^	−1.19 × 10^1^	−2.05 × 10^1^	−2.40 × 10^1^	−1.51 × 10^1^
	worst	−2.54 × 10^1^	−1.30 × 10^1^	−2.03 × 10^1^	−1.09 × 10^1^	−2.37 × 10^1^	−8.89 × 10^0^	−1.45 × 10^1^	−7.05 × 10^0^	−1.90 × 10^1^	−9.65 × 10^0^	−1.13 × 10^1^	−2.02 × 10^1^	−1.11 × 10^1^
	std	7.39 × 10^−1^	1.38 × 10^0^	5.91 × 10^−1^	5.09 × 10^−1^	9.65 × 10^−1^	2.98 × 10^0^	4.06 × 10^0^	1.64 × 10^0^	2.67 × 10^0^	9.90 × 10^−1^	4.42 × 10^0^	1.74 × 10^0^	2.01 × 10^0^
	median	−2.64 × 10^1^	−1.42 × 10^1^	−2.11 × 10^1^	−1.14 × 10^1^	−2.54 × 10^1^	−1.03 × 10^1^	−1.88 × 10^1^	−8.34 × 10^0^	−2.33 × 10^1^	−1.06 × 10^1^	−1.49 × 10^1^	−2.32 × 10^1^	−1.24 × 10^1^
	rank	1	8	5	10	2	11	6	13	4	12	7	3	9
C11-F3	mean	1.15 × 10^−5^	1.15 × 10^−5^	1.15 × 10^−5^	1.15 × 10^−5^	1.15 × 10^−5^	1.15 × 10^−5^	1.15 × 10^−5^	1.15 × 10^−5^	1.15 × 10^−5^	1.15 × 10^−5^	1.15 × 10^−5^	1.15 × 10^−5^	1.15 × 10^−5^
	best	1.15 × 10^−5^	1.15 × 10^−5^	1.15 × 10^−5^	1.15 × 10^−5^	1.15 × 10^−5^	1.15 × 10^−5^	1.15 × 10^−5^	1.15 × 10^−5^	1.15 × 10^−5^	1.15 × 10^−5^	1.15 × 10^−5^	1.15 × 10^−5^	1.15 × 10^−5^
	worst	1.15 × 10^−5^	1.15 × 10^−5^	1.15 × 10^−5^	1.15 × 10^−5^	1.15 × 10^−5^	1.15 × 10^−5^	1.15 × 10^−5^	1.15 × 10^−5^	1.15 × 10^−5^	1.15 × 10^−5^	1.15 × 10^−5^	1.15 × 10^−5^	1.15 × 10^−5^
	std	2.00 × 10^−19^	2.22 × 10^−11^	2.54 × 10^−9^	4.99 × 10^−11^	1.24 × 10^−15^	2.38 × 10^−14^	6.12 × 10^−19^	9.96 × 10^−13^	3.73 × 10^−15^	7.83 × 10^−14^	2.00 × 10^−19^	5.96 × 10^−20^	2.76 × 10^−18^
	median	1.15 × 10^−5^	1.15 × 10^−5^	1.15 × 10^−5^	1.15 × 10^−5^	1.15 × 10^−5^	1.15 × 10^−5^	1.15 × 10^−5^	1.15 × 10^−5^	1.15 × 10^−5^	1.15 × 10^−5^	1.15 × 10^−5^	1.15 × 10^−5^	1.15 × 10^−5^
	rank	1	11	13	12	6	8	4	10	7	9	3	2	5
C11-F4	mean	0.00 × 10^0^	0.00 × 10^0^	0.00 × 10^0^	0.00 × 10^0^	0.00 × 10^0^	0.00 × 10^0^	0.00 × 10^0^	0.00 × 10^0^	0.00 × 10^0^	0.00 × 10^0^	0.00 × 10^0^	0.00 × 10^0^	0.00 × 10^0^
	best	0.00 × 10^0^	0.00 × 10^0^	0.00 × 10^0^	0.00 × 10^0^	0.00 × 10^0^	0.00 × 10^0^	0.00 × 10^0^	0.00 × 10^0^	0.00 × 10^0^	0.00 × 10^0^	0.00 × 10^0^	0.00 × 10^0^	0.00 × 10^0^
	worst	0.00 × 10^0^	0.00 × 10^0^	0.00 × 10^0^	0.00 × 10^0^	0.00 × 10^0^	0.00 × 10^0^	0.00 × 10^0^	0.00 × 10^0^	0.00 × 10^0^	0.00 × 10^0^	0.00 × 10^0^	0.00 × 10^0^	0.00 × 10^0^
	std	0.00 × 10^0^	0.00 × 10^0^	0.00 × 10^0^	0.00 × 10^0^	0.00 × 10^0^	0.00 × 10^0^	0.00 × 10^0^	0.00 × 10^0^	0.00 × 10^0^	0.00 × 10^0^	0.00 × 10^0^	0.00 × 10^0^	0.00 × 10^0^
	median	0.00 × 10^0^	0.00 × 10^0^	0.00 × 10^0^	0.00 × 10^0^	0.00 × 10^0^	0.00 × 10^0^	0.00 × 10^0^	0.00 × 10^0^	0.00 × 10^0^	0.00 × 10^0^	0.00 × 10^0^	0.00 × 10^0^	0.00 × 10^0^
	rank	1	1	1	1	1	1	1	1	1	1	1	1	1
C11-F5	mean	−3.41 × 10^1^	−2.48 × 10^1^	−2.81 × 10^1^	−1.99 × 10^1^	−3.33 × 10^1^	−2.71 × 10^1^	−2.76 × 10^1^	−2.70 × 10^1^	−3.16 × 10^1^	−1.07 × 10^1^	−2.73 × 10^1^	−8.48 × 10^0^	−9.35 × 10^0^
	best	−3.47 × 10^1^	−2.59 × 10^1^	−2.92 × 10^1^	−2.21 × 10^1^	−3.39 × 10^1^	−3.15 × 10^1^	−2.78 × 10^1^	−3.17 × 10^1^	−3.42 × 10^1^	−1.28 × 10^1^	−3.15 × 10^1^	−1.21 × 10^1^	−1.08 × 10^1^
	worst	−3.34 × 10^1^	−2.38 × 10^1^	−2.76 × 10^1^	−1.75 × 10^1^	−3.19 × 10^1^	−2.18 × 10^1^	−2.72 × 10^1^	−2.45 × 10^1^	−2.75 × 10^1^	−9.02 × 10^0^	−2.42 × 10^1^	−6.75 × 10^0^	−7.67 × 10^0^
	std	5.90 × 10^−1^	9.52 × 10^−1^	7.70 × 10^−1^	2.52 × 10^0^	9.37 × 10^−1^	4.24 × 10^0^	2.83 × 10^−1^	3.55 × 10^0^	2.99 × 10^0^	1.69 × 10^0^	3.39 × 10^0^	2.63 × 10^0^	1.45 × 10^0^
	median	−3.42 × 10^1^	−2.47 × 10^1^	−2.78 × 10^1^	−2.00 × 10^1^	−3.36 × 10^1^	−2.76 × 10^1^	−2.77 × 10^1^	−2.58 × 10^1^	−3.23 × 10^1^	−1.04 × 10^1^	−2.68 × 10^1^	−7.55 × 10^0^	−9.46 × 10^0^
	rank	1	9	4	10	2	7	5	8	3	11	6	13	12
C11-F6	mean	−2.41 × 10^1^	−1.40 × 10^1^	−1.90 × 10^1^	−1.30 × 10^1^	−2.26 × 10^1^	−7.48 × 10^0^	−1.99 × 10^1^	−9.46 × 10^0^	−1.96 × 10^1^	−2.21 × 10^0^	−2.19 × 10^1^	−3.08 × 10^0^	−3.99 × 10^0^
	best	−2.74 × 10^1^	−1.46 × 10^1^	−2.04 × 10^1^	−1.37 × 10^1^	−2.58 × 10^1^	−1.65 × 10^1^	−2.30 × 10^1^	−1.74 × 10^1^	−2.24 × 10^1^	−2.51 × 10^0^	−2.66 × 10^1^	−6.00 × 10^0^	−9.24 × 10^0^
	worst	−2.30 × 10^1^	−1.38 × 10^1^	−1.72 × 10^1^	−1.20 × 10^1^	−2.13 × 10^1^	−4.20 × 10^0^	−1.29 × 10^1^	−2.11 × 10^0^	−1.80 × 10^1^	−2.11 × 10^0^	−1.78 × 10^1^	−2.11 × 10^0^	−2.11 × 10^0^
	std	2.32 × 10^0^	4.11 × 10^−1^	1.55 × 10^0^	8.23 × 10^−1^	2.23 × 10^0^	6.34 × 10^0^	5.02 × 10^0^	8.71 × 10^0^	2.22 × 10^0^	2.13 × 10^−1^	4.01 × 10^0^	2.04 × 10^0^	3.68 × 10^0^
	median	−2.30 × 10^1^	−1.38 × 10^1^	−1.92 × 10^1^	−1.32 × 10^1^	−2.17 × 10^1^	−4.60 × 10^0^	−2.19 × 10^1^	−9.16 × 10^0^	−1.91 × 10^1^	−2.11 × 10^0^	−2.16 × 10^1^	−2.11 × 10^0^	−2.31 × 10^0^
	rank	1	7	6	8	2	10	4	9	5	13	3	12	11
C11-F7	mean	8.61 × 10^−1^	1.61 × 10^0^	1.28 × 10^0^	1.92 × 10^0^	9.30 × 10^−1^	1.30 × 10^0^	1.74 × 10^0^	8.81 × 10^−1^	1.07 × 10^0^	1.72 × 10^0^	1.08 × 10^0^	1.12 × 10^0^	1.74 × 10^0^
	best	5.82 × 10^−1^	1.54 × 10^0^	1.14 × 10^0^	1.68 × 10^0^	7.57 × 10^−1^	1.13 × 10^0^	1.63 × 10^0^	8.18 × 10^−1^	8.15 × 10^−1^	1.53 × 10^0^	8.84 × 10^−1^	8.32 × 10^−1^	1.35 × 10^0^
	worst	1.03 × 10^0^	1.72 × 10^0^	1.43 × 10^0^	2.10 × 10^0^	1.01 × 10^0^	1.67 × 10^0^	1.92 × 10^0^	9.55 × 10^−1^	1.29 × 10^0^	1.86 × 10^0^	1.28 × 10^0^	1.37 × 10^0^	1.94 × 10^0^
	std	2.12 × 10^−1^	8.15 × 10^−2^	1.62 × 10^−1^	1.87 × 10^−1^	1.23 × 10^−1^	2.59 × 10^−1^	1.30 × 10^−1^	7.10 × 10^−2^	2.07 × 10^−1^	1.53 × 10^−1^	1.88 × 10^−1^	2.89 × 10^−1^	2.84 × 10^−1^
	median	9.18 × 10^−1^	1.58 × 10^0^	1.28 × 10^0^	1.95 × 10^0^	9.75 × 10^−1^	1.21 × 10^0^	1.71 × 10^0^	8.76 × 10^−1^	1.08 × 10^0^	1.74 × 10^0^	1.08 × 10^0^	1.15 × 10^0^	1.83 × 10^0^
	rank	1	9	7	13	3	8	12	2	4	10	5	6	11
C11-F8	mean	2.20 × 10^2^	2.85 × 10^2^	2.41 × 10^2^	3.26 × 10^2^	2.22 × 10^2^	2.57 × 10^2^	2.66 × 10^2^	2.24 × 10^2^	2.27 × 10^2^	2.24 × 10^2^	2.46 × 10^2^	4.70 × 10^2^	2.22 × 10^2^
	best	2.20 × 10^2^	2.59 × 10^2^	2.24 × 10^2^	2.85 × 10^2^	2.20 × 10^2^	2.20 × 10^2^	2.45 × 10^2^	2.20 × 10^2^	2.20 × 10^2^	2.20 × 10^2^	2.20 × 10^2^	2.48 × 10^2^	2.20 × 10^2^
	worst	2.20 × 10^2^	3.20 × 10^2^	2.57 × 10^2^	3.70 × 10^2^	2.25 × 10^2^	3.55 × 10^2^	3.12 × 10^2^	2.36 × 10^2^	2.35 × 10^2^	2.36 × 10^2^	2.94 × 10^2^	5.71 × 10^2^	2.30 × 10^2^
	std	0.00 × 10^0^	2.83 × 10^1^	1.53 × 10^1^	3.70 × 10^1^	2.98 × 10^0^	6.87 × 10^1^	3.26 × 10^1^	8.59 × 10^0^	8.93 × 10^0^	8.59 × 10^0^	3.67 × 10^1^	1.61 × 10^2^	5.25 × 10^0^
	median	2.20 × 10^2^	2.81 × 10^2^	2.41 × 10^2^	3.24 × 10^2^	2.22 × 10^2^	2.27 × 10^2^	2.54 × 10^2^	2.20 × 10^2^	2.27 × 10^2^	2.20 × 10^2^	2.36 × 10^2^	5.31 × 10^2^	2.20 × 10^2^
	rank	1	10	6	11	2	8	9	4	5	4	7	12	3
C11-F9	mean	8.79 × 10^3^	5.59 × 10^5^	3.80 × 10^5^	1.07 × 10^6^	2.02 × 10^4^	6.64 × 10^4^	3.76 × 10^5^	1.34 × 10^5^	4.31 × 10^4^	4.10 × 10^5^	8.26 × 10^5^	1.09 × 10^6^	1.95 × 10^6^
	best	5.46 × 10^3^	3.74 × 10^5^	3.36 × 10^5^	6.96 × 10^5^	1.10 × 10^4^	4.77 × 10^4^	2.08 × 10^5^	7.58 × 10^4^	1.85 × 10^4^	3.39 × 10^5^	7.07 × 10^5^	8.72 × 10^5^	1.87 × 10^6^
	worst	1.40 × 10^4^	6.43 × 10^5^	4.09 × 10^5^	1.25 × 10^6^	2.88 × 10^4^	8.44 × 10^4^	6.37 × 10^5^	2.03 × 10^5^	7.54 × 10^4^	5.26 × 10^5^	8.89 × 10^5^	1.33 × 10^6^	2.06 × 10^6^
	std	3.89 × 10^3^	1.33 × 10^5^	3.36 × 10^4^	2.64 × 10^5^	8.26 × 10^3^	1.64 × 10^4^	2.06 × 10^5^	5.50 × 10^4^	2.53 × 10^4^	8.65 × 10^4^	8.53 × 10^4^	2.58 × 10^5^	1.01 × 10^5^
	median	7.83 × 10^3^	6.10 × 10^5^	3.87 × 10^5^	1.16 × 10^6^	2.06 × 10^4^	6.68 × 10^4^	3.29 × 10^5^	1.28 × 10^5^	3.93 × 10^4^	3.87 × 10^5^	8.54 × 10^5^	1.07 × 10^6^	1.93 × 10^6^
	rank	1	9	7	11	2	4	6	5	3	8	10	12	13
C11-F10	mean	−2.15 × 10^1^	−1.40 × 10^1^	−1.69 × 10^1^	−1.23 × 10^1^	−1.90 × 10^1^	−1.44 × 10^1^	−1.29 × 10^1^	−1.47 × 10^1^	−1.41 × 10^1^	−1.13 × 10^1^	−1.31 × 10^1^	−1.14 × 10^1^	−1.11 × 10^1^
	best	−2.18 × 10^1^	−1.52 × 10^1^	−1.71 × 10^1^	−1.26 × 10^1^	−1.94 × 10^1^	−1.89 × 10^1^	−1.35 × 10^1^	−2.12 × 10^1^	−1.46 × 10^1^	−1.14 × 10^1^	−1.37 × 10^1^	−1.14 × 10^1^	−1.11 × 10^1^
	worst	−2.08 × 10^1^	−1.33 × 10^1^	−1.65 × 10^1^	−1.20 × 10^1^	−1.86 × 10^1^	−1.20 × 10^1^	−1.24 × 10^1^	−1.14 × 10^1^	−1.29 × 10^1^	−1.12 × 10^1^	−1.24 × 10^1^	−1.13 × 10^1^	−1.10 × 10^1^
	std	4.99 × 10^−1^	8.71 × 10^−1^	2.77 × 10^−1^	3.01 × 10^−1^	4.21 × 10^−1^	3.24 × 10^0^	5.10 × 10^−1^	4.62 × 10^0^	8.26 × 10^−1^	8.59 × 10^−2^	6.66 × 10^−1^	4.09 × 10^−2^	5.64 × 10^−2^
	median	−2.17 × 10^1^	−1.37 × 10^1^	−1.70 × 10^1^	−1.22 × 10^1^	−1.90 × 10^1^	−1.33 × 10^1^	−1.28 × 10^1^	−1.31 × 10^1^	−1.44 × 10^1^	−1.13 × 10^1^	−1.33 × 10^1^	−1.14 × 10^1^	−1.11 × 10^1^
	rank	1	7	3	10	2	5	9	4	6	12	8	11	13
C11-F11	mean	5.72 × 10^5^	5.81 × 10^6^	9.92 × 10^5^	8.88 × 10^6^	1.66 × 10^6^	5.96 × 10^6^	1.22 × 10^6^	1.31 × 10^6^	3.84 × 10^6^	5.22 × 10^6^	1.41 × 10^6^	5.23 × 10^6^	6.14 × 10^6^
	best	2.61 × 10^5^	5.54 × 10^6^	7.73 × 10^5^	8.58 × 10^6^	1.55 × 10^6^	4.96 × 10^6^	1.11 × 10^6^	6.12 × 10^5^	3.65 × 10^6^	5.19 × 10^6^	1.27 × 10^6^	5.21 × 10^6^	6.09 × 10^6^
	worst	8.29 × 10^5^	6.18 × 10^6^	1.17 × 10^6^	9.07 × 10^6^	1.80 × 10^6^	7.20 × 10^6^	1.38 × 10^6^	2.74 × 10^6^	4.20 × 10^6^	5.24 × 10^6^	1.59 × 10^6^	5.25 × 10^6^	6.21 × 10^6^
	std	2.61 × 10^5^	3.12 × 10^5^	1.83 × 10^5^	2.18 × 10^5^	1.26 × 10^5^	9.74 × 10^5^	1.22 × 10^5^	1.01 × 10^6^	2.59 × 10^5^	2.39 × 10^4^	1.40 × 10^5^	2.19 × 10^4^	5.34 × 10^4^
	median	5.99 × 10^5^	5.77 × 10^6^	1.01 × 10^6^	8.93 × 10^6^	1.65 × 10^6^	5.83 × 10^6^	1.19 × 10^6^	9.45 × 10^5^	3.76 × 10^6^	5.22 × 10^6^	1.40 × 10^6^	5.23 × 10^6^	6.12 × 10^6^
	rank	1	10	2	13	6	11	3	4	7	8	5	9	12
C11-F12	mean	1.20 × 10^6^	8.41 × 10^6^	3.38 × 10^6^	1.33 × 10^7^	1.27 × 10^6^	5.04 × 10^6^	5.82 × 10^6^	1.33 × 10^6^	1.42 × 10^6^	1.44 × 10^7^	5.80 × 10^6^	2.32 × 10^6^	1.45 × 10^7^
	best	1.16 × 10^6^	8.06 × 10^6^	3.28 × 10^6^	1.23 × 10^7^	1.20 × 10^6^	4.77 × 10^6^	5.41 × 10^6^	1.18 × 10^6^	1.26 × 10^6^	1.35 × 10^7^	5.51 × 10^6^	2.15 × 10^6^	1.44 × 10^7^
	worst	1.25 × 10^6^	8.71 × 10^6^	3.45 × 10^6^	1.41 × 10^7^	1.35 × 10^6^	5.18 × 10^6^	6.03 × 10^6^	1.47 × 10^6^	1.56 × 10^6^	1.50 × 10^7^	6.01 × 10^6^	2.53 × 10^6^	1.47 × 10^7^
	std	4.72 × 10^4^	2.86 × 10^5^	7.84 × 10^4^	7.65 × 10^5^	7.14 × 10^4^	2.02 × 10^5^	3.04 × 10^5^	1.27 × 10^5^	1.32 × 10^5^	6.60 × 10^5^	2.26 × 10^5^	1.65 × 10^5^	1.11 × 10^5^
	median	1.20 × 10^6^	8.43 × 10^6^	3.40 × 10^6^	1.33 × 10^7^	1.27 × 10^6^	5.10 × 10^6^	5.93 × 10^6^	1.33 × 10^6^	1.44 × 10^6^	1.45 × 10^7^	5.84 × 10^6^	2.30 × 10^6^	1.45 × 10^7^
	rank	1	10	6	11	2	7	9	3	4	12	8	5	13
C11-F13	mean	1.54 × 10^4^	1.59 × 10^4^	1.54 × 10^4^	1.63 × 10^4^	1.55 × 10^4^	1.55 × 10^4^	1.55 × 10^4^	1.55 × 10^4^	1.55 × 10^4^	1.59 × 10^4^	1.29 × 10^5^	1.55 × 10^4^	3.00 × 10^4^
	best	1.54 × 10^4^	1.57 × 10^4^	1.54 × 10^4^	1.59 × 10^4^	1.55 × 10^4^	1.55 × 10^4^	1.55 × 10^4^	1.55 × 10^4^	1.55 × 10^4^	1.56 × 10^4^	9.30 × 10^4^	1.55 × 10^4^	1.55 × 10^4^
	worst	1.54 × 10^4^	1.63 × 10^4^	1.54 × 10^4^	1.73 × 10^4^	1.55 × 10^4^	1.55 × 10^4^	1.56 × 10^4^	1.55 × 10^4^	1.55 × 10^4^	1.65 × 10^4^	1.77 × 10^5^	1.55 × 10^4^	7.34 × 10^4^
	std	9.09 × 10^−3^	3.19 × 10^2^	9.34 × 10^−1^	7.32 × 10^2^	2.94 × 10^0^	1.17 × 10^1^	5.03 × 10^1^	2.87 × 10^1^	8.83 × 10^0^	4.14 × 10^2^	3.98 × 10^4^	2.59 × 10^1^	3.04 × 10^4^
	median	1.54 × 10^4^	1.57 × 10^4^	1.54 × 10^4^	1.60 × 10^4^	1.55 × 10^4^	1.55 × 10^4^	1.55 × 10^4^	1.55 × 10^4^	1.55 × 10^4^	1.58 × 10^4^	1.22 × 10^5^	1.55 × 10^4^	1.56 × 10^4^
	rank	1	9	2	11	3	4	8	7	6	10	13	5	12
C11-F14	mean	1.83 × 10^4^	1.13 × 10^5^	1.85 × 10^4^	2.30 × 10^5^	1.86 × 10^4^	1.95 × 10^4^	1.92 × 10^4^	1.94 × 10^4^	1.92 × 10^4^	3.12 × 10^5^	1.91 × 10^4^	1.91 × 10^4^	1.91 × 10^4^
	best	1.82 × 10^4^	8.58 × 10^4^	1.84 × 10^4^	1.69 × 10^5^	1.85 × 10^4^	1.93 × 10^4^	1.91 × 10^4^	1.93 × 10^4^	1.91 × 10^4^	3.03 × 10^4^	1.88 × 10^4^	1.90 × 10^4^	1.88 × 10^4^
	worst	1.84 × 10^4^	1.58 × 10^5^	1.86 × 10^4^	3.31 × 10^5^	1.87 × 10^4^	2.01 × 10^4^	1.93 × 10^4^	1.95 × 10^4^	1.94 × 10^4^	6.02 × 10^5^	1.93 × 10^4^	1.93 × 10^4^	1.94 × 10^4^
	std	7.16 × 10^1^	3.38 × 10^4^	1.06 × 10^2^	7.62 × 10^4^	7.26 × 10^1^	3.89 × 10^2^	1.33 × 10^2^	8.11 × 10^1^	1.54 × 10^2^	2.88 × 10^5^	2.28 × 10^2^	1.34 × 10^2^	2.51 × 10^2^
	median	1.83 × 10^4^	1.04 × 10^5^	1.85 × 10^4^	2.09 × 10^5^	1.86 × 10^4^	1.94 × 10^4^	1.92 × 10^4^	1.94 × 10^4^	1.92 × 10^4^	3.07 × 10^5^	1.91 × 10^4^	1.91 × 10^4^	1.91 × 10^4^
	rank	1	11	2	12	3	10	7	9	8	13	4	6	5
C11-F15	mean	3.29 × 10^4^	9.11 × 10^5^	1.08 × 10^5^	1.92 × 10^6^	3.29 × 10^4^	5.46 × 10^4^	2.19 × 10^5^	3.31 × 10^4^	3.31 × 10^4^	1.55 × 10^7^	3.01 × 10^5^	3.33 × 10^4^	7.97 × 10^6^
	best	3.28 × 10^4^	3.76 × 10^5^	4.32 × 10^4^	8.04 × 10^5^	3.29 × 10^4^	3.30 × 10^4^	3.30 × 10^4^	3.30 × 10^4^	3.30 × 10^4^	3.24 × 10^6^	2.66 × 10^5^	3.33 × 10^4^	3.63 × 10^6^
	worst	3.30 × 10^4^	2.29 × 10^6^	1.81 × 10^5^	5.02 × 10^6^	3.30 × 10^4^	1.19 × 10^5^	3.14 × 10^5^	3.32 × 10^4^	3.31 × 10^4^	2.31 × 10^7^	3.24 × 10^5^	3.33 × 10^4^	1.37 × 10^7^
	std	7.69 × 10^1^	9.71 × 10^5^	7.77 × 10^4^	2.17 × 10^6^	6.40 × 10^1^	4.52 × 10^4^	1.33 × 10^5^	6.65 × 10^1^	4.91 × 10^1^	9.48 × 10^6^	2.85 × 10^4^	8.68 × 10^0^	4.83 × 10^6^
	median	3.29 × 10^4^	4.89 × 10^5^	1.04 × 10^5^	9.33 × 10^5^	3.30 × 10^4^	3.32 × 10^4^	2.65 × 10^5^	3.31 × 10^4^	3.31 × 10^4^	1.78 × 10^7^	3.06 × 10^5^	3.33 × 10^4^	7.29 × 10^6^
	rank	1	10	7	11	2	6	8	4	3	13	9	5	12
C11-F16	mean	1.34 × 10^5^	9.48 × 10^5^	1.35 × 10^5^	1.96 × 10^6^	1.38 × 10^5^	1.45 × 10^5^	1.42 × 10^5^	1.42 × 10^5^	1.46 × 10^5^	8.92 × 10^7^	1.88 × 10^7^	7.99 × 10^7^	7.67 × 10^7^
	best	1.31 × 10^5^	2.89 × 10^5^	1.34 × 10^5^	4.75 × 10^5^	1.36 × 10^5^	1.42 × 10^5^	1.36 × 10^5^	1.33 × 10^5^	1.43 × 10^5^	8.70 × 10^7^	9.54 × 10^6^	6.61 × 10^7^	6.20 × 10^7^
	worst	1.36 × 10^5^	2.24 × 10^6^	1.36 × 10^5^	4.86 × 10^6^	1.41 × 10^5^	1.47 × 10^5^	1.48 × 10^5^	1.51 × 10^5^	1.51 × 10^5^	9.18 × 10^7^	3.40 × 10^7^	9.54 × 10^7^	9.81 × 10^7^
	std	2.39 × 10^3^	9.22 × 10^5^	1.07 × 10^3^	2.07 × 10^6^	2.71 × 10^3^	2.41 × 10^3^	4.91 × 10^3^	7.70 × 10^3^	3.93 × 10^3^	2.14 × 10^6^	1.11 × 10^7^	1.33 × 10^7^	1.61 × 10^7^
	median	1.33 × 10^5^	6.31 × 10^5^	1.36 × 10^5^	1.24 × 10^6^	1.37 × 10^5^	1.46 × 10^5^	1.42 × 10^5^	1.42 × 10^5^	1.44 × 10^5^	8.91 × 10^7^	1.58 × 10^7^	7.90 × 10^7^	7.33 × 10^7^
	rank	1	8	2	9	3	6	5	4	7	13	10	12	11
C11-F17	mean	1.93 × 10^6^	8.99 × 10^9^	2.32 × 10^9^	1.56 × 10^+10^	2.29 × 10^6^	1.29 × 10^9^	9.73 × 10^9^	3.12 × 10^6^	3.02 × 10^6^	2.24 × 10^+10^	1.13 × 10^+10^	2.09 × 10^+10^	2.19 × 10^+10^
	best	1.92 × 10^6^	7.66 × 10^9^	2.11 × 10^9^	1.12 × 10^+10^	1.96 × 10^6^	1.06 × 10^9^	6.94 × 10^9^	2.30 × 10^6^	2.04 × 10^6^	2.15 × 10^+10^	9.90 × 10^9^	1.85 × 10^+10^	2.05 × 10^+10^
	worst	1.94 × 10^6^	9.97 × 10^9^	2.54 × 10^9^	1.90 × 10^+10^	2.91 × 10^6^	1.47 × 10^9^	1.29 × 10^+10^	3.75 × 10^6^	4.89 × 10^6^	2.34 × 10^+10^	1.19 × 10^+10^	2.42 × 10^+10^	2.48 × 10^+10^
	std	1.20 × 10^4^	1.07 × 10^9^	2.00 × 10^8^	3.54 × 10^9^	4.49 × 10^5^	2.22 × 10^8^	2.65 × 10^9^	7.04 × 10^5^	1.35 × 10^6^	7.94 × 10^8^	9.64 × 10^8^	2.71 × 10^9^	2.04 × 10^9^
	median	1.92 × 10^6^	9.17 × 10^9^	2.32 × 10^9^	1.60 × 10^+10^	2.15 × 10^6^	1.31 × 10^9^	9.52 × 10^9^	3.21 × 10^6^	2.58 × 10^6^	2.23 × 10^+10^	1.16 × 10^+10^	2.05 × 10^+10^	2.12 × 10^+10^
	rank	1	7	6	10	2	5	8	4	3	13	9	11	12
C11-F18	mean	9.42 × 10^5^	5.52 × 10^7^	6.58 × 10^6^	1.19 × 10^8^	9.72 × 10^5^	2.05 × 10^6^	9.61 × 10^6^	9.88 × 10^5^	1.03 × 10^6^	3.11 × 10^7^	1.12 × 10^7^	1.35 × 10^8^	1.15 × 10^8^
	best	9.38 × 10^5^	3.80 × 10^7^	3.95 × 10^6^	8.21 × 10^7^	9.50 × 10^5^	1.80 × 10^6^	4.13 × 10^6^	9.64 × 10^5^	9.67 × 10^5^	2.47 × 10^7^	8.33 × 10^6^	1.14 × 10^8^	1.11 × 10^8^
	worst	9.45 × 10^5^	6.28 × 10^7^	1.13 × 10^7^	1.36 × 10^8^	1.03 × 10^6^	2.40 × 10^6^	1.69 × 10^7^	1.00 × 10^6^	1.20 × 10^6^	3.37 × 10^7^	1.41 × 10^7^	1.50 × 10^8^	1.19 × 10^8^
	std	2.77 × 10^3^	1.22 × 10^7^	3.59 × 10^6^	2.64 × 10^7^	4.11 × 10^4^	3.05 × 10^5^	5.66 × 10^6^	1.73 × 10^4^	1.19 × 10^5^	4.54 × 10^6^	2.70 × 10^6^	1.72 × 10^7^	3.63 × 10^6^
	median	9.43 × 10^5^	6.00 × 10^7^	5.54 × 10^6^	1.29 × 10^8^	9.54 × 10^5^	2.01 × 10^6^	8.72 × 10^6^	9.95 × 10^5^	9.79 × 10^5^	3.31 × 10^7^	1.11 × 10^7^	1.39 × 10^8^	1.15 × 10^8^
	rank	1	10	6	12	2	5	7	3	4	9	8	13	11
C11-F19	mean	1.03 × 10^6^	5.43 × 10^7^	6.68 × 10^6^	1.16 × 10^8^	1.14 × 10^6^	2.47 × 10^6^	1.03 × 10^7^	1.48 × 10^6^	1.36 × 10^6^	3.57 × 10^7^	6.29 × 10^6^	1.73 × 10^8^	1.15 × 10^8^
	best	9.68 × 10^5^	4.64 × 10^7^	6.10 × 10^6^	1.01 × 10^8^	1.07 × 10^6^	2.23 × 10^6^	2.06 × 10^6^	1.13 × 10^6^	1.23 × 10^6^	2.50 × 10^7^	2.39 × 10^6^	1.57 × 10^8^	1.12 × 10^8^
	worst	1.17 × 10^6^	6.91 × 10^7^	8.09 × 10^6^	1.46 × 10^8^	1.29 × 10^6^	2.92 × 10^6^	1.86 × 10^7^	1.96 × 10^6^	1.55 × 10^6^	4.46 × 10^7^	8.26 × 10^6^	2.00 × 10^8^	1.19 × 10^8^
	std	9.97 × 10^4^	1.08 × 10^7^	9.97 × 10^5^	2.24 × 10^7^	1.10 × 10^5^	3.21 × 10^5^	8.17 × 10^6^	3.67 × 10^5^	1.38 × 10^5^	8.90 × 10^6^	2.80 × 10^6^	1.97 × 10^7^	2.72 × 10^6^
	median	9.83 × 10^5^	5.09 × 10^7^	6.26 × 10^6^	1.09 × 10^8^	1.10 × 10^6^	2.37 × 10^6^	1.02 × 10^7^	1.41 × 10^6^	1.34 × 10^6^	3.67 × 10^7^	7.25 × 10^6^	1.68 × 10^8^	1.15 × 10^8^
	rank	1	10	7	12	2	5	8	4	3	9	6	13	11
C11-F20	mean	9.41 × 10^5^	5.78 × 10^7^	5.91 × 10^6^	1.26 × 10^8^	9.60 × 10^5^	1.83 × 10^6^	7.30 × 10^6^	9.73 × 10^5^	9.99 × 10^5^	3.47 × 10^7^	1.43 × 10^7^	1.60 × 10^8^	1.16 × 10^8^
	best	9.36 × 10^5^	5.08 × 10^7^	5.21 × 10^6^	1.10 × 10^8^	9.57 × 10^5^	1.65 × 10^6^	6.88 × 10^6^	9.63 × 10^5^	9.78 × 10^5^	3.39 × 10^7^	9.51 × 10^6^	1.46 × 10^8^	1.10 × 10^8^
	worst	9.47 × 10^5^	6.84 × 10^7^	6.66 × 10^6^	1.50 × 10^8^	9.62 × 10^5^	2.14 × 10^6^	7.87 × 10^6^	9.84 × 10^5^	1.02 × 10^6^	3.55 × 10^7^	2.22 × 10^7^	1.74 × 10^8^	1.20 × 10^8^
	std	5.01 × 10^3^	7.87 × 10^6^	6.32 × 10^5^	1.77 × 10^7^	2.43 × 10^3^	2.45 × 10^5^	4.43 × 10^5^	9.87 × 10^3^	1.70 × 10^4^	6.93 × 10^5^	5.81 × 10^6^	1.61 × 10^7^	4.36 × 10^6^
	median	9.41 × 10^5^	5.59 × 10^7^	5.89 × 10^6^	1.22 × 10^8^	9.61 × 10^5^	1.77 × 10^6^	7.23 × 10^6^	9.72 × 10^5^	1.00 × 10^6^	3.47 × 10^7^	1.28 × 10^7^	1.60 × 10^8^	1.16 × 10^8^
	rank	1	10	6	12	2	5	7	3	4	9	8	13	11
C11-F21	mean	1.27 × 10^1^	5.04 × 10^1^	2.17 × 10^1^	7.67 × 10^1^	1.59 × 10^1^	2.99 × 10^1^	3.89 × 10^1^	2.76 × 10^1^	2.24 × 10^1^	1.01 × 10^2^	4.08 × 10^1^	1.06 × 10^2^	1.03 × 10^2^
	best	9.97 × 10^0^	4.15 × 10^1^	2.03 × 10^1^	5.71 × 10^1^	1.38 × 10^1^	2.65 × 10^1^	3.56 × 10^1^	2.45 × 10^1^	2.06 × 10^1^	4.85 × 10^1^	3.59 × 10^1^	9.17 × 10^1^	5.90 × 10^1^
	worst	1.50 × 10^1^	6.00 × 10^1^	2.35 × 10^1^	9.64 × 10^1^	1.82 × 10^1^	3.15 × 10^1^	4.30 × 10^1^	3.06 × 10^1^	2.48 × 10^1^	1.49 × 10^2^	4.37 × 10^1^	1.18 × 10^2^	1.26 × 10^2^
	std	2.41 × 10^0^	8.39 × 10^0^	1.42 × 10^0^	1.82 × 10^1^	2.18 × 10^0^	2.41 × 10^0^	3.46 × 10^0^	3.63 × 10^0^	1.93 × 10^0^	4.32 × 10^1^	3.68 × 10^0^	1.36 × 10^1^	3.27 × 10^1^
	median	1.30 × 10^1^	5.01 × 10^1^	2.14 × 10^1^	7.67 × 10^1^	1.59 × 10^1^	3.08 × 10^1^	3.85 × 10^1^	2.76 × 10^1^	2.21 × 10^1^	1.03 × 10^2^	4.18 × 10^1^	1.07 × 10^2^	1.14 × 10^2^
	rank	1	9	3	10	2	6	7	5	4	11	8	13	12
C11-F22	mean	1.61 × 10^1^	4.69 × 10^1^	2.75 × 10^1^	6.37 × 10^1^	1.91 × 10^1^	3.22 × 10^1^	4.65 × 10^1^	3.24 × 10^1^	2.50 × 10^1^	1.03 × 10^2^	4.68 × 10^1^	1.07 × 10^2^	9.29 × 10^1^
	best	1.15 × 10^1^	4.08 × 10^1^	2.22 × 10^1^	4.61 × 10^1^	1.62 × 10^1^	2.82 × 10^1^	4.01 × 10^1^	2.48 × 10^1^	2.39 × 10^1^	6.66 × 10^1^	3.91 × 10^1^	8.97 × 10^1^	9.20 × 10^1^
	worst	1.96 × 10^1^	5.25 × 10^1^	3.28 × 10^1^	7.33 × 10^1^	2.13 × 10^1^	3.48 × 10^1^	5.12 × 10^1^	3.75 × 10^1^	2.59 × 10^1^	1.22 × 10^2^	5.58 × 10^1^	1.18 × 10^2^	9.45 × 10^1^
	std	4.20 × 10^0^	5.30 × 10^0^	5.25 × 10^0^	1.27 × 10^1^	2.54 × 10^0^	3.00 × 10^0^	5.26 × 10^0^	5.93 × 10^0^	9.26 × 10^−1^	2.62 × 10^1^	7.23 × 10^0^	1.35 × 10^1^	1.17 × 10^0^
	median	1.67 × 10^1^	4.73 × 10^1^	2.75 × 10^1^	6.76 × 10^1^	1.94 × 10^1^	3.30 × 10^1^	4.72 × 10^1^	3.36 × 10^1^	2.52 × 10^1^	1.12 × 10^2^	4.62 × 10^1^	1.10 × 10^2^	9.26 × 10^1^
	rank	1	9	4	10	2	5	7	6	3	12	8	13	11
Sum rank		22	191	109	231	55	146	145	118	97	222	157	198	224
Mean rank		1.00 × 10^0^	8.68 × 10^0^	4.95 × 10^0^	1.05 × 10^1^	2.50 × 10^0^	6.64 × 10^0^	6.59 × 10^0^	5.36 × 10^0^	4.41 × 10^0^	1.01 × 10^1^	7.14 × 10^0^	9.00 × 10^0^	1.02 × 10^1^
Total rank		1	2	12	4	13	3	11	9	6	7	10	5	8
Wilcoxon: *p*-value			1.71 × 10^−15^	9.77 × 10^−15^	1.71 × 10^−15^	7.10 × 10^−15^	3.66 × 10^−15^	1.71 × 10^−15^	3.99 × 10^−12^	7.10 × 10^−15^	5.36 × 10^−15^	8.52 × 10^−15^	2.54 × 10^−15^	5.36 × 10^−15^

## Data Availability

Not applicable.
